# The Age of Phage: Friend or Foe in the New Dawn of Therapeutic and Biocontrol Applications?

**DOI:** 10.3390/ph14030199

**Published:** 2021-02-28

**Authors:** Ahmad Y. Hassan, Janet T. Lin, Nicole Ricker, Hany Anany

**Affiliations:** 1 Guelph Research and Development Centre, Agriculture and Agri-Food Canada, Guelph, ON N1G 5C9, Canada; ahassa10@uoguelph.ca; 2 Department of Molecular and Cellular Biology, College of Biological Science, University of Guelph, Guelph, ON N1G 2W1, Canada; janet.lin@canada.ca; 3 Department of Pathobiology, Ontario Veterinary College, University of Guelph, Guelph, ON N1G 2W1, Canada; nricker@uoguelph.ca; 4 Department of Food Science, Ontario Agricultural College, University of Guelph, Guelph, ON N1G 2W1, Canada

**Keywords:** bacteriophage, phage therapy, biocontrol, transduction, antibiotic resistance

## Abstract

Extended overuse and misuse of antibiotics and other antibacterial agents has resulted in an antimicrobial resistance crisis. Bacteriophages, viruses that infect bacteria, have emerged as a legitimate alternative antibacterial agent with a wide scope of applications which continue to be discovered and refined. However, the potential of some bacteriophages to aid in the acquisition, maintenance, and dissemination of negatively associated bacterial genes, including resistance and virulence genes, through transduction is of concern and requires deeper understanding in order to be properly addressed. In particular, their ability to interact with mobile genetic elements such as plasmids, genomic islands, and integrative conjugative elements (ICEs) enables bacteriophages to contribute greatly to bacterial evolution. Nonetheless, bacteriophages have the potential to be used as therapeutic and biocontrol agents within medical, agricultural, and food processing settings, against bacteria in both planktonic and biofilm environments. Additionally, bacteriophages have been deployed in developing rapid, sensitive, and specific biosensors for various bacterial targets. Intriguingly, their bioengineering capabilities show great promise in improving their adaptability and effectiveness as biocontrol and detection tools. This review aims to provide a balanced perspective on bacteriophages by outlining advantages, challenges, and future steps needed in order to boost their therapeutic and biocontrol potential, while also providing insight on their potential role in contributing to bacterial evolution and survival.

## 1. Introduction

The use of antibiotics to treat a wide range of infections, saving millions of lives and revolutionizing the field of medicine since their discovery, has enabled them to be one of the most impactful scientific discoveries in modern history. However, the resulting consequences of their extensive use has signified a new era for medicine. The rapid rise of antibacterial resistance in bacteria across the globe, coupled with declines in the development of novel antibacterial agents, has resulted in the need for new approaches in combating bacterial infections [1]. Bacteriophages (phages), viruses that infect bacteria, have emerged as a viable alternative to the declining utility of traditional antimicrobials to mitigate the risk of pathogenic bacteria [2]. Their ubiquity in nature, versatility, and innate resiliency has elevated their status from mere research organisms to potentially viable and necessary tools in the fight against rising antimicrobial resistance [3].

While their potential utility and benefits continued to be examined and documented, one of the key hurdles facing the progression and ultimate acceptance of bacteriophages for therapeutic and biocontrol purposes is their ability to contribute to the horizontal transfer of genes, which can have negative consequences. The transfer of resistance, virulence, and other negatively associated genes via bacteriophages is a major area in which further examination and understanding is needed, so that proper steps may be taken to minimize its potentially harmful impacts.

Conversely, bacteriophages provide an incredible sense of versatility for potential application. The use of whole phage particles and phage components continues to be studied, as well as their use across a variety of industries and purposes. The application of bacteriophages within medical, agricultural, and food processing settings provides some of the most promising opportunities for their regulatory approval and commercialization [4,5]. Their ability and potential efficacy to be used against bacteria in both planktonic and biofilm settings provide another reason for their continued examination [6]. Phages continue to be studied as not only bacterial killers, but also as potential active bacterial detectors, and while understanding surrounding them remains relatively limited, computational and bioengineering advancements have the potential to further bolster the utility of the bacterial viruses.

**Figure 1:**
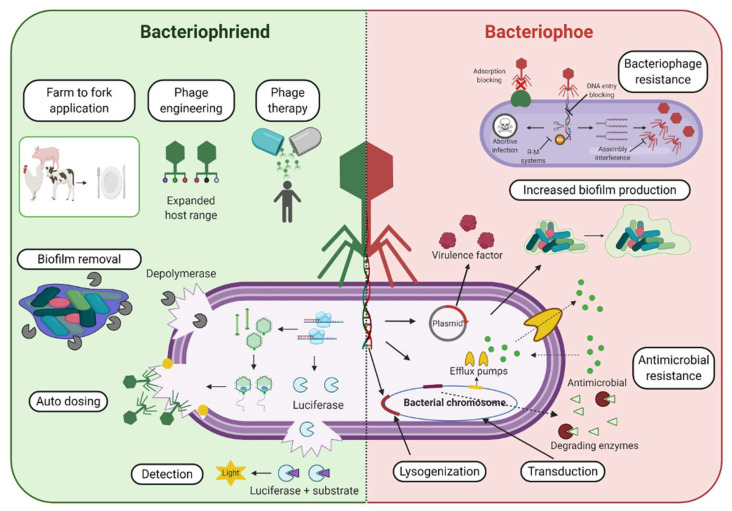
Bacteriophages and the Antimicrobial Resistance Crisis: Friend or Foe? An overview of various phage-based applications and examples that display the potential benefit and “friend” aspect of bacteriophages (**left**, green background side) in combatting antimicrobial resistant bacterial strains, as well as some potentially detrimental or negatively associated outcomes that can contribute to the “foe” aspect of phage-based use (**right**, red background side) and their role in the acquisition, maintenance, and dissemination of antibacterial resistance genes.

Therefore, we aim to provide a balanced perspective on bacteriophages in this review (Figure 1), outlining the current advantages, challenges, and future steps needed in order to boost their therapeutic and biocontrol potential, while also providing insight into their role in the acquisition, maintenance, and dissemination of genes that may benefit bacterial survival through transduction.

## 2. Bacterial Antimicrobial Resistance—An Ongoing Crisis

### 2.1. The Boom of Antibacterial Agents

Evidence exists showing the use of various substances for their antimicrobial properties since ancient times [7,8]. However, the scientific discovery and characterization of compounds throughout the 20th century [9], particularly antibiotics, revolutionized civilization with direct and substantial benefits to global health and the global economy [10]. The significance of the contributions of antibacterial agents to the control of infectious diseases, previously among the leading causes of human morbidity and mortality for most of human existence [10], cannot be emphasized enough. The golden era of antimicrobials, spanning the 1940s to the 1970s [11], was highlighted by the discovery and development of numerous antibiotic drug classes [10]. Bacterial illnesses, such as pneumonia and tuberculosis, had accounted for 22% of all deaths in the United States of America in 1930, with this figure declining to a mere 6% by 1952 [12] following the availability of antibiotics. While approximately 280,000–300,000 Americans died annually as a result of bacterial infections throughout 1930–1936 [12], fewer than 95,000 died in 1952 from similar causes [12]. The therapeutic use of antibiotics for the treatment of severe infections, as well as their prophylactic use in certain clinical situations, has proven to be powerful and crucial for the betterment of human health [13]. Nonetheless, a major consequence of the expanded use of antibacterial agents has been the heightened presence of resistant organisms as a result of the increase in evolutionary selective pressures [14].

### 2.2. The Rise of Antibacterial Resistance

Studies have shown the relationship shared between the emergence and dissemination of antimicrobial resistant bacterial strains with increased antibiotic consumption [15]. Through natural selection, the presence of antibacterial agents removes sensitive competitors and allows resilient bacteria to remain and reproduce, thus increasing their numbers [16]. The lack of regulations in many countries worldwide regarding antibiotic prescription and use for human and in agriculture means that they are easily accessible, plentiful, and cheap, all components which promote their overuse [17]. Findings that 30–50% of recorded antibiotic prescriptions include incorrect treatment indication, choice of agent, or duration of therapy indicate a great degree of misuse within clinical circumstances [15,18]. The incorrect prescription of antibiotics has questionable therapeutic benefits and compounds selection for drug-resistant bacterial strains, while further exposing patients to potential complications [19]. Additionally, suboptimal dosing of antibiotics can promote resistance by supporting genetic alterations through mutagenesis and horizontal gene transfer [20]. Poor patient compliance throughout the course of antibacterial therapies also presents a source of worry regarding the progression of antibacterial resistance, with an adherence study conducted by Llor et al., 2013, reporting excellent adherence to antibiotic treatment by nearly 30% of patients, while close to 25% of patients displayed non-adherence to the antibiotic treatment of respiratory infections [21]. Moreover, poor antimicrobial stewardship practices within healthcare settings lead to an increase in the development and transmission of resistant bacterial infections within hospitals [22]. While increases in patient–patient interactions and the excessive use of antibiotics contribute to the selection of drug-resistant bacterial strains within clinical environments; increased regulations regarding hygiene and sanitation, particularly handwashing and hand gloving, have been noted to be most effective in minimizing transmission [23]. Crucially, limiting the spread of antibacterial resistance requires a two-prong approach; limiting the development of resistance through the controlled use of antibacterial agents and limiting transmission through proper sanitation and management of residues [24,25]. 

One of the key contributors to the development of resistant bacterial strains is the continuous use of antimicrobials for agricultural purposes, specifically relating to food-producing animals [26]. The extent of their use for the purposes of growth promotion, feed efficiency improvement, and disease prophylaxis contributes heavily to the potential dangers they pose to human health due to the likelihood of them contributing to the transfer of antimicrobial-resistant pathogens to the human population [27]. On an annual basis in the United States, 80% of the total 17 million kilograms of antibiotics consumed each year are used on food-producing animals [10]. The intricate web of interactions shared among animals, humans, and the environment necessitates a holistic, One Health approach to address the rise of antibacterial resistance within these three sectors of life [28]. The presence of antibacterial resistance among prominent zoonotic pathogens such as *E. coli*, *Salmonella* spp., *Campylobacter* spp., and *Listeria* spp. provides a worrisome obstacle in the food-producing animal industry [29], with high rates of resistance being found in the gastrointestinal tracts and carcasses of food-producing animals [30]. The presence of resistant foodborne pathogens may lead to outbreaks of resistant disease upon the consumption of contaminated meat products. Additionally, the use of antibacterial agents in agriculture can also have negative impacts on the environmental microbiome [31]. Up to 90% of livestock-consumed antibiotics are excreted as waste in stool or urine [32], which can then be broadly dispersed through groundwater, surface runoff, and fertilizer [15]. This further exposes microorganisms within the environment to growth-inhibiting agents and alters the surrounding ecology by increasing the proportion of resistant versus vulnerable bacteria [33]. Without being addressed, this problem can become cyclical and accumulative within the microbiome over time, leading to further negative impacts as resistant bacteria grow in number and strength [34,35]. Thus, the use of antibacterial agents in food-producing animals is a major force behind the rise in the prevalence of antimicrobial resistance.

### 2.3. The Sobering Reality of Antibacterial Resistance

A vital step in minimizing the impact of antibacterial resistance lies in the bettered understanding of the mechanisms of antibacterial action and their corresponding resistance mechanisms so that novel approaches can be undertaken to lessen its ramifications. In general, a variety of antibacterial agents have been used to combat microbial growth, including antibiotics, antiseptics, and disinfectants [36]. Antibiotics, defined as selectively toxic natural or synthetic organic substances which destroy or inhibit bacteria and other microorganisms [36], are considered the primary threat behind rising antibacterial resistance. Antibiotics are commonly classified into five modes of action [37]: (1) inhibition of cell wall biosynthesis, (2) inhibition of membrane function, (3) inhibition of protein biosynthesis, (4) inhibition of nucleic acid synthesis, and (5) antimetabolites. 

While the bulk of our understanding and use is related to antibiotics, antibacterial agents such as antiseptics, defined as biocides which destroy or inhibit microbial growth in or on living tissues, and disinfectants, defined as biocides used on surfaces or inanimate objects [36], have received increased scrutiny regarding their connection to antimicrobial resistance, as well as their varying levels of toxicity to their environment [36,38]. Some of their mechanisms of action include DNA strand breakage, inhibition of DNA synthesis, damaging of the cytoplasmic membrane, cross-linking of macromolecules, and the uncoupling of enzymes [36]. While antiseptics and disinfectants have been used thoroughly as bactericides, an increasing number of reports have shown their association with growing bacterial resistance. For example, the widespread use of triclosan, a bisphenol compound that is commonly used in hand soaps and household items for its antimicrobial properties [39,40], has been found to lead to various bacterial strains becoming resistant to it, such as *Salmonella enterica* [41] and *E. coli* [42]. Alarmingly, environmental exposure to triclosan has been noted to increase occurrences of cross-resistance to unrelated antibiotics, particularly chloramphenicol and tetracycline, which may exasperate the severe public health threat of burgeoning multidrug resistant bacteria in environmental microbial communities [39]. As such, the incorporation of triclosan into household soap products and over-the-counter health products was banned by the United States Food and Drug Administration (FDA) in 2016 [43]. Stable resistance to another common disinfectant and antiseptic, chlorhexidine diacetate, has been observed in *Pseudomonas aeruginosa* [42]. Furthermore, silver compounds, which have been used in combination with antibiotics to boost their efficacy, have been shown to enhance the selection of antibiotic resistant genes among bacteria [44]. Additionally, bacterial resistance has been reported to quaternary ammonium compounds (QACs) which are widely used as disinfectants in both medical and food-processing environments [45]. For example, nearly 13% of staphylococci isolates and 30% of *Pseudomonas* isolates exhibited an ability to grow in concentrations of benzalkonium chloride which are normally recommended for disinfection purposes [46].

Mechanisms associated with antibacterial resistance are often grouped into the following categories [47,48]: (1) Prevention of cellular uptake or efflux, where bacteria inhibit the accumulation of a drug or actively transport it out of the cell to prevent the drug from reaching its cellular target; (2) drug inactivation or modification, where resistance genes code for enzymes that chemically modify and inactivate a drug through hydrolysis; (3) target overproduction or enzymatic bypass, where bacteria increase production of a targeted enzyme in order to ensure there is a sufficient amount of antibacterial-free enzyme to carry out normal enzymatic reactions, or to find ways to bypass the need for the functional target enzyme; (4) target modification, where mutations can lead to structural changes in targets that render select antibacterial agents ineffective; and (5) target mimicry, where bacteria produce certain proteins that bind and isolate drugs, thus preventing them from binding to their targets. The breadth of mechanisms employed by bacteria to resist the effects of various antibacterial agents provides a challenge regarding future approaches aimed to combat the antimicrobial resistance crisis and prevent its widening reach.

The increasing number of findings regarding the wide variety of antibacterial agents to which bacteria may become resistant to provides a worrisome development for a variety of industries. As a result, mortality rates due to resistant bacterial infections have been on the rise, with around 25,000 patients in the EU dying from them annually [9]. In the United States, an estimated 2 million patients a year deal with infections resulting from drug-resistant bacteria, with more than 63,000 passing from hospital-acquired infections [9,10]. Moreover, costs associated with extra healthcare costs and productivity losses resulting from resistant bacterial infections in the EU are estimated to be worth at least 1.5 billion EUR [49]. These indirect and direct costs are further exasperated in the United States where they reach annual estimates of 55 billion USD [50]. On its present trajectory, the antimicrobial resistance crisis has the potential to reach levels of 10 million deaths worldwide by 2050 and costs of up to 100 trillion dollars [10,50], unprecedented levels which may far exceed the impacts observed within the pre-antibacterial era. Therefore, a host of scientific, regulatory, and economic measures are urgently needed in order to address this pressing crisis and mitigate its future impacts.

## 3. Bacteriophage Biology and History

Since their independent discovery over a century ago by Frederick Twort and Felix d’Herelle, bacteriophages have played a key role as model organisms, helping further the development of fields such as molecular biology, microbial genetics, biodiversity, and medicine [51]. They have been used by Salvador Luria and Max Delbruck to observe the spontaneous and random nature of genetic mutations within bacteria [52], by Alfred Hershey and Martha Chase to confirm the role of DNA and not protein as the hereditary material of life [53], by Joshua Lederberg and Norton Zinder to discover transduction, the process in which viruses transfer DNA between different cells [54], as well as by Francis Crick, Leslie Barnett, Sydney Brenner, and R.J. Watts-Tobin to demonstrate how nucleotides are read three base pairs at a time as codons to represent individual amino acids [55]. Furthermore, the discovery and characterization of the bacterial CRISPR-Cas adaptive immunity systems has opened a new world of applications, among them the possibilities of synthetic biology and genome editing [56,57,58]. Its potential applications to the biomedical and agricultural sectors, among others, provide another example of the key role bacteriophages have played within biology. Additionally, phages have played an important role in bettering our understanding of global biodiversity, as the combination of their ubiquity throughout nature and advancements in omics-based analyses has given scientists insight into the role phages play within the environment relating to microbial turnover, gene transfer, metabolic reprogramming, and biogeochemical cycling [59,60]. While phage therapy research was hindered between the 1940s and early 1980s due to antibiotics being the antibacterial of choice for therapeutic purposes within Western countries [61,62], the continued use and development of bacteriophages as a viable option for use within Eastern countries, particularly those within the former Soviet Union [63,64], displayed the promising potential of phages. In light of the declining utility of antibiotics and rise in antibacterial resistance, a renaissance of interest in phage research has sparked worldwide.

Bacteriophages are often noted as the most abundant biological entity on earth, with estimates of their population being in the range of 10^31^ viral particles across the biosphere [65]. As with other viruses, phages are infectious particles composed of a minimum of two components, (1) protein subunits that form a protective capsid surrounding (2) nucleic acids composing the phage genome [66]. The capsid has three main roles within a phage’s life cycle [67]: (1) to protect the phage genome (e.g., from nucleic acid-degrading enzymes), (2) effecting phage adsorption to susceptible bacteria, and (3) delivery of the phage genome into the cytoplasm of an infected bacterium. Phages genomes may be comprised of double-stranded or single-stranded DNA, as well as double-stranded or single-stranded RNA [68]. Phage genome vary in size from the extremely small genomes of the Leviviridae family of RNA phages which can be as small as 3.3 kb in length [69], to the comparatively larger genome of the *Bacillus megaterium* phage G with a genome that is 497 kb in length [70], as well as reported Lak megaphages with genome sizes in the range of ~540 to 552 kb [71].

Phages are traditionally categorized in regard to their morphology in structures such as tailed, polyhedral (icosahedral or quasi-icosahedral bodies), filamentous, or pleomorphic [72]. The morphology of bacteriophages is closely related to the complexity of their genome, as a larger genome often indicates a large capsid and therefore a more complex organization [68]. Interestingly, it has been noted that through the analysis of 5568 bacteriophages by electron microscopy, 96.2% were observed to be tailed and the remaining 3.7% as polyhedral, filamentous, or pleomorphic [73]. Hollow tubes within tailed phages act as pipes during infection that ensure the secure transfer of nucleic acids into a host cell [68]. At the end of a phage’s tail is an adsorption apparatus that acts as a special adhesive system which serves to recognize host cells and penetrate their wall [68].

A key aspect in the biology of bacteriophages is their replication cycles, identified as the lytic cycle and the lysogenic cycle [74,75]. Phages that exclusively undergo the lytic cycle, also known as the productive or virulent cycle, are termed as lytic or virulent [76]. Virulent phages infect bacterial hosts, overtake cellular metabolic functions, and subvert them to the production of phage progeny, whereupon bacterial host death occurs upon lysis of the bacterium and release of newly formed phage particles [77]. Additionally, the number of phage progeny produced which spread and infect other cells is dependent on the type of phage [78]. The model lytic cycle consists of five sequential stages: adsorption, penetration, maturation, assembly, and lysis [67].

Adsorption begins when specialized structures such as spikes or fibers bind to specific surface molecules (e.g., lipopolysaccharides and OmpC proteins) on the target bacterium [67,79]. Bacterial receptors vary among strains and they may be located on the cell wall, plasma membrane, flagella, pili, or capsules [80]. In addition, phage adsorption is reliant on a variety of physical and chemical interactions within and surrounding the phage–bacteria complex [67,81]. Next, the bacteriophage uses a variety of mechanisms and enzymes to degrade the cell wall of the host, enabling the outer and inner membranes to be punctured and the viral genome to be injected into the bacterium’s cytoplasm [82]. While the viral DNA or RNA is often the only component which enters the host, filamentous DNA phages such as that of *E. coli* (e.g., f1, and fd) reportedly enter the inner membrane of the host cell’s envelope while being coated, upon which the protein coat disassembles into subunits and subsequently releases the DNA intracellularly [83]. Following entry of the viral genome into the bacterial host, the expression of “early proteins”, which are needed to replicate the phage genome and to further modify present cellular machinery, begins as a way to divert the synthetic capacity of the cell towards phage progeny production [67,84]. Numerous copies of the phage genome are synthesized at this time which can be used for the transcription and translation of late proteins, which make up several components of the tail assembly [67,85]. At this stage within the lytic cycle, phage components are packaged and assembled into newly formed virions, occurring either spontaneously or with the aid of specific enzymes [67]. Copies of the phage genome are inserted into preassembled protein shells termed procapsids, and this assembly involves complex interaction between specific scaffolding and head structure proteins [86]. In the final step of the lytic cycle, progeny phage particles are released with cell lysis. Specific enzymes such as lysin and holin break down the cell membrane from within and liberate the new infectious phages that are capable of continuing the cycle over again within new susceptible host cells [87]. 

Select bacteriophages may employ the lysogenic cycle, where their nucleic acid is incorporated into the genome of a host bacterium or separately as an extrachromosomal plasmid [88], leading to its presence as a prophage within the bacterium and its progeny [89]. The prophage may be carried for several generations by the lysogenic bacterial host until it is induced into the lytic cycle to produce phage progeny as described in the previous paragraph. Induction can be spontaneous, but usually occurs following adverse environmental conditions, including changes in nutrition, pH, temperature or other external stressors, which can trigger the cell’s DNA damage response [90]. Details regarding lysogeny and its potential impact on the utility of bacteriophages in general will be covered in greater detail in the following section.

## 4. Bacteriophage Contribution to the Evolution and Mobilization of Antimicrobial Resistance

In response to selective pressures such as the presence of antibacterial agents, bacteria acquire new genetic traits through mutations and horizontal gene transfer (HGT), which in turn provide potential selective advantages for the survival of bacteria. Mutations, often accompanied with decreases in an organism’s fitness [91], occur relatively slowly at an average mutation rate of 10^−6^ to 10^−9^ per nucleotide per generation [92]. Conversely, HGT offers bacteria a diverse array of transferrable genetic elements that can enable bacteria to adapt and respond to stresses more rapidly through the acquisition of large DNA sequences in single transfers, ranging from 1 to more than 100 kb [92]. Estimates of 10^25^ phage infections initiating every second worldwide [93] and up to 20% of the bacterial genome being composed of viral origin indicate the potentially large impact which HGT can have on bacterial evolution [94]. Bacteriophages have been observed to provide contributions to bacteria through the transfer of DNA sequences including chromosomal sequences such as prophages, and mobile genetic elements such as transposons, plasmids, pathogenicity islands, and insertion elements [95]. They are more often involved in genome diversification in intraspecies alignments than in interspecies scenarios due to their innate host specificity [92]. As such, it is of interest to understand the extent to which bacteriophages aid in the transfer of genetic material across bacterial hosts, and in particular, their role in propagating antibacterial resistance, bacterial virulence, and pathogenicity [96].

### 4.1. Fundamentals of Bacteriophage-Mediated Gene Transfer

One of the primary ways in which bacteriophages impact bacterial fitness is by introducing new fitness factors to their bacterial hosts through transduction. A mechanism of horizontal gene transfer, transduction is noted as the transfer of bacterial DNA between a bacteriophage-infected bacterium and a bacteriophage-susceptible bacterium [97]. Its role in the transfer of genetic material within natural environments has traditionally been underestimated, although metagenomic analysis of viromes has suggested that functional bacterial genes may exist in up to 60% of bacteriophages, displaying their potential as reservoirs of bacterial diversity [95]. Transduction is regarded as a primary driving force behind microbial evolution [98] with estimates of 20 × 10^15^ gene transfer events per second [99]. Additionally, prophages are believed to be present in nearly half of sequenced bacterial genomes [100]. Traditionally, two types of transduction are observed, generalized and specialized. Generalized transduction occurs when phage packaging accidentally incorporates bacterial DNA instead of phage DNA through faulty genome packaging, resulting in the transfer of any host gene across hosts [98]. Generalized transduction occurs in the lytic cycle of a bacteriophage and in around 1 in 10,000 phage progeny [101]. In generalized transduction, viral DNA is not present within the transducing particle and it is the transduced bacterial DNA which is present [102]. Viruses such as those of the genus *Viunalikevirus*, capable of efficient generalized transduction, may present as useful agents for synthetic biology and functional genomics studies, and continue to be investigated for their potential ecological significance [103,104]. Conversely, specialized transduction, which has been investigated extensively through study of phage P22 [105,106,107,108], is viewed as a property of temperate phages and occurs due to the imprecise excision of an integrated prophage genome from the bacterial chromosome, leading to adjacent pieces of bacterial DNA being incorporated into new phage virions during packaging [101]. In specialized transduction, both viral DNA and the transduced bacterial DNA are present, although there are relatively smaller quantities of bacterial DNA transferred compared to generalized transduction [102]. 

**Figure 2:**
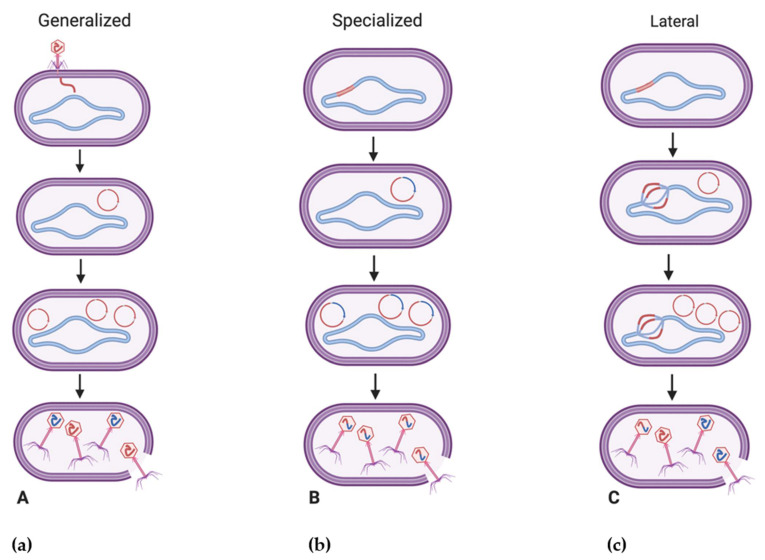
Bacteriophage-mediated transduction. Simplified depiction of generalized (**a**), specialized (**b**), and lateral transduction (**c**). Viral genome is represented in red while the bacterial genome is represented in blue. Note the varying presence of viral and bacterial genetic material differing across the three mechanisms of phage-mediated transduction. Adapted from [98].

While generalized and specialized transduction are believed to occur as a result of erroneous phage processes, the recent characterization of lateral transduction (Figure 2), which has only been described in *Staphylococcus aureus* temperate phages to date, may point to this mechanism as being a natural part of the phage life cycle [98,109]. Believed to transfer significantly larger amounts of genetic material at frequencies at least 1000 times greater than generalized or specialized transduction [98], lateral transduction is observed to occur upon the delayed excision of a prophage where bacteriophages may initiate DNA replication while still integrated in the host genome, resulting in multiple prophage genomes being present [110]. Following this, headful packaging and assembly may initiate on some genomes and lead to the transfer of viral and bacterial chromosomal DNA to other bacteria, while other phage genomes may simultaneously continue with normal phage maturation [110]. Due to its efficient transfer of several hundred kilobases of genetic material [110], it is believed that lateral transduction is a leading force in the rapid evolution of bacteria as opposed to the comparatively smaller amounts of genetic material (~40 kb) transferred through generalized transduction, for example [96,111].

It is important to note that transduction would be an inconsequential process if it did not provide benefits to both bacteria and bacteriophages. Observations of the competitive advantages which lysogens have over prophage-lacking competitors supports the notion that the presence of prophages may function as a mutualistic trait [111,112]. Once considered as bacterial parasites which silently persist within bacteria, prophages are now understood to have a symbiotic relationship with their bacterial hosts [113]. As bacterial viruses, the obligate dependence of phages on bacteria for functions relating to energy production and biosynthetic activities means that by providing their bacterial hosts with access to genes encoding for a variety of fitness advantages, the growth rate and physiology of the bacterial host may be improved through the integration of the prophage, which may then lead to growth in phage population numbers and dissemination to different environment and hosts [92,111,114].

### 4.2. Bacteriophage Contributions to Antibacterial Resistance

In an attempt to understand how to minimize the spread of antibacterial resistance, recent findings have shown the significant contributions of phage-mediated transduction in propagating antibacterial resistance genes. In particular, documented cases of phages propagating resistance genes in clinically relevant bacterial pathogens listed as part of the World Health Organization’s Priority Pathogens List (Table 1) pose a significant barrier for the advancement of bacteriophages as a potential biocontrol agent [115]. While prophages may be recognized as vehicles and environmental reservoirs for antimicrobial resistance genes [116,117,118], their net effects on bacterial fitness and relevance to the evolution of resistant bacterial pathogens are yet to be fully understood. Specifically, as active prophages may excise to form progeny phage particles which increase the mobility of carried virulence and resistance genes; the presence of cryptic prophages, considered as relatively permanent reservoirs of virulence, resistance, and tolerance genes due to their inability to form active phage particles to lyse their hosts, further laments the beneficial impact which prophages may have on bacterial survival [119]. Moreover, insight into the prevalence of virulence factors and antimicrobial resistance genes encoded by prophages in different strains of seven bacterial pathogens may suggest differing distribution patterns, with prophage-encoded antimicrobial resistance genes being detected in a broader range of hosts than prophages containing virulence factors, which were conserved in only a couple species [120]. However, more analysis is needed using more strains isolated from different clinical and natural environments before drawing a generalized conclusion from these observations. Mechanisms contributing to phage-encoded virulence factors include improvements in gene mobility resulting from increased virulence factor presence among bacterial populations, as well as epistatic interactions between virulence and phage genes that subsequently enhance the utility of virulence factors to bacterial hosts, among others [114]. Nonetheless, it is important to note that the extent to which resistance genes are functionally encoded for among phages may be significantly less than portrayed through bioinformatic analysis, hinting that the presence of resistance genes among phage genomes may be more conservative in nature and stressing the need for further investigation [121].

Studies have observed the transfer of antibiotic resistance genes in critical pathogens such as *Acinetobacter baumannii* and *Pseudomonas aeruginosa*. Regions around 30 kb in size, including genes associated with resistance to aminoglycosides (*armA*), β-lactam (*bla*_TEM-1_), tetracycline (*tet*(B)), and nalidixic acid (*gyrA*-81L), were found to be transduced to an antibiotic-susceptible *A. baumannii* 17,978 strain from an original multidrug-resistant *A. baumannii* NU-60 strain [122]. Recent genomic analysis has exhibited the extent of prophage presence across the *A. baumannii* genome, with 78% of observed intact prophages conferring presumed virulence factors [123]. Furthermore, low-frequency transduction to imipenem and high-frequency transduction to cefotaxime, ceftazidime, and aztreonam has been observed in vitro with *P. aeruginosa* [124]. In addition, phage-mediated transduction of numerous genes encoding resistance to "last-resort antibiotics" such as fluoroquinolones (*qnrB19* and *qnrS2*), carbapenems (*bla*_KPC-2_, *bla*_KPC-3_, *bla*_BKC-1_, and *bla*_OXA-656_), and colistin (*mcr-4* and *mcr-5*) on small plasmids has been observed, indicating the potentially drastic effects bacteriophages can have on the dissemination of antibacterial resistance genes [125]. Findings have also displayed how transduction could be a major contributor to the emergence and spread of antibacterial resistance in clinically relevant *Staphylococcus aureus* [126]. For example, plasmids providing resistance against tetracycline and penicillin have been noted to be transduced between *S. aureus* isolates [127]. Transduction of large sections of the Staphylococcal Cassette Chromosome *mec* (SCC*mec*) alongside the methicillin resistance gene *mecA* has also been observed [128].

Bacteriophages have also been noted to be involved in the transduction of antibacterial resistance genes in common foodborne pathogens listed as part of the World Health Organization’s Priority Pathogens List (Table 1) [129]. Infection of *E. coli* by Stx-converting phage, modified to incorporate resistance genes for tetracycline or chloramphenicol, resulted in transductants exhibiting resistance to the antibiotics [130]. In addition, 24.7% of 243 tested coliphages originating from retail chicken meat samples were observed to transduce one or more resistance gene encoding for tetracycline, ampicillin, kanamycin, and chloramphenicol to a laboratory strain of *E. coli* [131]. Transduction of antibiotic resistance has been primarily reported in laboratory strains, where the efficient transfer of resistance genes to tetracycline was observed to occur from *E. coli* 0157:H7 to *E. coli* K-12 [132]. Furthermore, transduction of antibiotic resistance genes has also been observed in *Salmonella*-infecting phages, particularly P22-like phages [129]. Transduction of genes conferring resistance to ampicillin (*amp*), chloramphenicol (*cam*), and tetracycline (*tet*) was observed between donor and recipient strains of *Salmonella* Typhimurium DT104 strains [133]. The same group also observed that 14 of 16 transductants were able to co-transduce genes conferring resistance to sulfonamides (*sul*) and streptomycin (*str*), along with the previously mentioned *amp*, *cam*, and *tet* genes. More recently, genome scanning has displayed the common presence of P22-like prophages in 18 *Salmonella* serovars, hinting that generalized transduction may be more prevalent than previously believed [129,134].

**Table 1:** Observed phage-mediated transduction events involving antibiotic resistance genes among bacterial pathogens identified as part of the World Health Organization’s Priority Pathogens since 2010.

Bacterial Pathogen	Phage	Resistance Gene	Antibiotic	Reference
*Acinetobacter baumannii*	Unknown	*arm*A *bla*_TEM-1_ *tet*(B) *gyrA*-81L	Aminoglycoside resistance Β-Lactamase resistance Tetracycline resistance Nalidixic Acid resistance	[122]
*Acinetobacter baumannii*	Unknown	*bla*_NDM-1_	Β-Lactamase resistance	[135]
*Pseudomonas aeruginosa*	Unknown	*bla*_VIM_ *bla*_TEM_ *mecA* *qnrA* *qnrS*	B-Lactamase resistance Methicillin resistance Quinolone resistance	[136]
*Staphylococcus aureus*	Φ19	*erm*(C)	Erythromycin resistance	[137]
*Staphylococcus aureus*	Φ20	*erm*(C)	Erythromycin resistance	[137]
*Staphylococcus aureus*	80α	*erm*(C)	Erythromycin resistance	[137]
*Staphylococcus aureus*	Φ52A	tetK cadD blaZ	Β-Lactamase resistance	[127]
*Staphylococcus aureus*	Φ80α	*tet*K *cad*D *bla*Z	Tetracycline resistance	[127]
*Staphylococcus aureus*	Φ29	*tet*K	Tetracycline resistance	[127]
*Escherichia coli*	933W	*tet*(A)	Tetracycline resistance	[132]
*Escherichia coli*	Various	*bla*_TEM_ *floR* *aphA1* *tet*(A)	Ampicillin resistance Chloramphenicol resistance Kanamycin resistance Tetracycline resistance	[131]
*Escherichia coli*	Unknown	*qnr*A *qnr*S	Quinolone resistance	[138]
*Escherichia coli*	Unknown	*bla*_TEM_ *bla*_CTX-M9_	Β-Lactamase resistance	[139]
*Escherichia coli*	Unknown	*sul1* *armA* *bla*_TEM_ *bla*_CTX-M-1_ *bla*_CTX-M-9_ *bla*_OXA-48_ bla_VIM_ *qnrA* *qnrS*	Sulfonamide resistance B-Lactamase resistance Quinolone resistance	[140]
*Escherichia coli*	Unknown	*bla*_TEM_ *bla*_CTX-M-9_ *bla*_VIM_ *qnrA* *qnrS*	B-Lactamase resistance Quinolone resistance	[141]
*Salmonella enterica*	SJ46	*bla_CTX-M_*	B-Lactamase resistance	[142]
*Salmonella enterica*	Unknown	Unknown	Kanamycin resistance	[143]
*Salmonella enterica*	Unknown	*tet*G	Tetracycline resistance	[134]
*Salmonella enterica*	ΦEB49	*ΔlacZ::* *kan*	Kanamycin resistance	[144]
*Salmonella enterica*	ΦEB47	*ΔlacZ::* *kan*	Kanamycin resistance	[144]
*Salmonella enterica*	ΦEB32	*ΔlacZ::* *kan*	Kanamycin resistance	[144]
*Salmonella enterica*	ΦEB5	*ΔlacZ::* *kan*	Kanamycin resistance	[144]
*Enterococcus faecium*	NG_048231.1	*tetM*	Tetracycline resistance	[142]
*Enterococcus faecium*	EFRM31	GEN	Gentamicin resistance	[145]

### 4.3. Additional Contributions of Bacteriphages to Bacterial Virulence

In addition to the phage-mediated distribution of antibacterial resistance genes, bacteriophages also impact bacterial fitness in a variety of other ways. Phages have been noted to play a role in the emergence of pathogens, with two distinguishable ways in which prophages may encode for virulence factors. The first observed situation is when a pathogen depends on a specific prophage-encoded toxin to cause disease, as in the case with *Vibrio cholerae*, Shiga toxin-producing *Escherichia coli*, *Corynebacterium diphtheriae*, and *Clostridium botulinum.* For example, an outbreak of enteroaggregative hemorrhagic *E. coli* O104:H4 that led to 3,842 human infections was reported to have gained virulence through the uptake of a Stx2a virulence marker by way of a Shiga toxin-encoding bacteriophage [146]. More recently, whole-genome sequencing-based epidemiological analysis has indicated the horizontal transfer of the *sopE* virulence gene via temperate bacteriophage mTmV, leading to the emergence and clonal distribution of a new epidemic *S. enterica* Typhimurium clone [147]. The second scenario involves certain pathogenic bacteria which harbor a multitude of prophages, with each phage-encoded virulence factor contributing a small amount to the overall pathogenicity of the lysogen [92]. Some pathogens which follow this second model include *Staphylococcus aureus*, *Streptococcus pyogenes*, and *Salmonella enterica* serovar Typhimurium [92].

The presence of prophages within a bacterial chromosome can naturally lead to their lysogen hosts benefitting of protection from further infection by other bacteriophages, termed superinfection immunity [148]. Following prophage establishment, superinfection immunity may also be transmitted to many bacterial generations as well [149]. These developments are in line with the symbiotic relationship shared among prophages existing in bacterial cells and is another way which enables their continued survival. For example, superinfection exclusion systems such as that of the P22 phage are considered to be of benefit to the life cycle of the particular virus as a result of the protection they afford [150]. Mechanisms by which superinfection immunity is achieved relate to the prevention of phage binding or phage genome injection through the bacterial envelope, as well as through the blocking of phage genomes from passing through the cellular envelope following them passing through outer membrane modifications [113]. For example, superinfection immunity has been observed in *Salmonella* phage P22 (sieA) [151] and *Vibrio cholerae* phage K139 (Orf2) [152]. While superinfection immunity may be beneficial in protecting bacteria against closely related bacteriophages, its benefits are believed to be limited due to the natural diversity of bacteriophage populations [78]. Recent studies have thoroughly examined the prevalence of prophage-mediated superinfection immunity, as well as the mechanisms which contribute to it [148].

### 4.4. Further Considerations—Co-Evolution and Interactions between Bacteriophages and Other Mobile Genetic Elements

There are a number of important interactions between bacteriophages and other mobile genetic elements, including plasmids, genomic islands, and integrative conjugative elements (ICEs) that contribute to bacterial evolution. One of the most important elements which enables bacteriophages to have such an impact on their bacterial hosts is the presence of phage-encoded integrase enzymes which enable the unidirectional and highly specific recombination of foreign DNA into the bacterial genome [153]. Two distinct families of phage integrases, tyrosine recombinases and serine recombinases, mediate the efficient recombination of short phage DNA strands at the phage attachment site (*att*P) and bacterial DNA at the bacterial attachment site (*att*B) [154]. Serine recombinases have been found to be larger in size while recognizing shorter *att*P sequences and not requiring host cofactors [153]. In comparison, tyrosine recombinases have been found to recognize and mediate the strand cleavage of longer *att*P sequences, while commonly also requiring other proteins encoded by the phage or host bacteria [153]. Tyrosine recombinases can be further divided into sub-families based on C-terminal catalytic domains [155], which fall into two broad categories. The first is simple recombinases that are more closely related to the XerC/D recombinases necessary for ensuring chromosomal dimer segregation [156]. The simple recombinases include a diversity of functions [157], including phase variation of pili by invertases and the acquisition of antibiotic resistance genes by class 1 integrons, and there are a small number of prophage that also utilize a simple recombinase, as identified for the Brujita Mycobacteriophage [158]. The second major group of tyrosine recombinases differ from the simple recombinases due to the presence of an N-terminal arm-binding domain [155,159]. These arm-binding tyrosine recombinases are common to both bacteriophages and other mobile genetic elements such as genomic islands and ICEs [157,160]. Notably, related integrase genes targeting the same insertion site can be found in both bacteriophage and ICEs including those within the lambda and P4 integrase families [161] that all target tRNA genes. Similarly, the SXT integrase lineage of ICEs [162] and Enterobacterial cdt1 phage have related integrases targeting the *prfC* gene. 

Although previous work has suggested that phage and genomic island integrases evolved separately [163], large scale analyses of the tyrosine recombinases support a shared evolution [157]. The modular nature of bacteriophages and ICEs may explain the relatedness observed between these diverse mobile genetic elements, as recombination events that impact transfer mechanisms can occur independently of the integration functions [164]. The functional equivalence of the integration module for phage and ICE movement has recently been demonstrated in *Bacillus subtilis* [165]. Similarly, ICEs with different conjugation machineries have been associated with closely related integrases [166]. As noted above, there have also been reports of ICEs and phage targeting the same chromosomal locations [167], which would provide opportunities for recombination and co-evolution of ICEs and phage over evolutionary time [160]. 

The existence of “phage-like plasmids” also known as “circular plasmid prophages” has been known since the discovery of the P1 phage, but additional examples of elements combining the features of plasmids and phage were rare until recently [168]. These combinations highlight the interconnected nature of mobile element categories, which provides an evolutionary benefit through genetic flexibility [169]. The plasmids from the incompatibility group identified as IncY are also members of the P1 phage group and have recently been associated with important antimicrobial resistance genes including *bla*_CTX-M-15_ and *mcr-1* [170]. Several members of the P1 phage group have also been identified as integrated components in larger multi-drug resistant plasmids, although the functionality of these bacteriophage has not been determined [168]. Similarly, the co-occurrence of one lineage of phage-like plasmids (D6-like plasmids) have recently been found to co-occur with multi-drug resistant IncC plasmids in *Salmonella* Typhimurium ST213 although a functional association between the two mobile elements has not been established [171].

Recently a new family of mobile elements has been described and categorized as phage-inducible chromosomal islands (PICI) [172]. Like the other mobile elements described in this section, PICI also encode a phage integrase and excisionase and have now been reported in both Gram-positive and Gram-negative bacteria, illustrating their potential importance in bacterial evolution. The characterization of phage-inducible chromosomal islands has renewed interest in the biosynthetic applications of phage integrases [173]. 

In addition to how integrases may contribute to the unfavorable impacts of bacteriophages on bacterial host survival, their versatility and efficiency has warranted investigation in regard to their potential applications and utility as tools in synthetic biology, which has been reviewed in previous articles [153,154]. While bacteriophages have been found to contribute to bacterial fitness through the transfer of genes associated with resistance and virulence, transduction as a mechanism of horizontal gene transfer is largely associated with temperate phages following the lysogenic cycle [174]. Despite the various studies that have displayed the mutualistic interactions between bacteriophages and their hosts [111], and described how lysogeny provides phages with direct benefits when bacterial hosts are scarce but resources are plentiful [175], few studies have examined the incidence of phage-related sequences in bacterial genomes, with present estimates accounting for these elements to take up around 10% of bacterial genomes [176]. Nonetheless, a temperate phage’s “decision” to integrate its genome into the bacterial chromosome of its host as a prophage is merely a choice for its continued survival, owing to its obligate dependence on its bacterial host [111]. In comparison, the ability of virulent bacteriophages to employ the lytic viral cycle, ultimately leading to the production of numerous phage progeny, provides phages with a great deal of potential for use as novel antibacterial agents, of which their applications will be discussed in subsequent sections.

## 5. The Unintended Invitation to Revisit Bacteriophages—Phages as a Tool in Biocontrol and Therapy to Combat Antimicrobial Resistance

### 5.1. Phage Selection Criteria for Biocontrol and Therapy

Bacteriophages must meet several parameters in order to ensure their safety and efficacy before they can be used for biocontrol and therapeutic purposes. Firstly, it is accepted that selected phages must be virulent (strictly lytic), thus lacking the ability to lysogenize targeted hosts [177]. This can help to minimize the transduction potential of the infecting bacteriophage, including the transfer of genes associated with resistance and virulence as discussed in the previous section. The lifestyle of a phage and the presence of potentially dangerous genetic determinants may be analyzed through the use of microbiological techniques by testing their ability to transfer selected markers between bacterial strains, through PCR-based techniques such as testing for the presence of bacterial DNA in phage particles, and through the use of genome sequencing and bioinformatic predictions in order to search for specific sequences associated with gene integration and toxin production [6]. Interestingly, advances in sequencing capabilities and synthetic biology techniques have led to innovative approaches incorporating the use of temperate phages and their lytic variants in phage therapy [178]. While strictly lytic phages will likely be the agent of choice in coming years, these advances highlight the potential viability and value of using temperate phages, including taking advantage of their natural ability for genome integration to either directly kill targeted bacteria through interference with host metabolism, or by rendering them as less pathogenic [178].

Moreover, since a high number of therapeutic phages ( at least 1 × 10^8^ plaque-forming units (PFU)/mL) must be used in order to ensure sufficient contact and rapid infection of targeted cells [67], selected phages should be easily propagated in liquid media with high titer. The host range of selected phages must also be considered, with all epidemiologically important strains of a target bacterium being covered, as some bacterial strains contain several serovars which need to be managed [67]. An optimal balance between too narrow of a host range (which could lead to some strains of the same species not being affected) and too broad of a host range (which could lead to the killing of beneficial bacteria present) must be found [6]. While phages used for therapeutic purposes may follow a “personalized medicine” approach, where pathogenic strains are identified within an individual and specific phages are then selected from pre-existing banks for treatment [179], using phage cocktails, where phages of different specificities are utilized in therapeutic applications, is recommended [6]. The continuous arms race proceeding between bacteriophages and their bacterial hosts relates to the close relationship shared between bacteria and bacteriophages, where bacteria are under constant pressure from their viral invaders, and thus seek to gain resistance to phages to prolong their survival. While being the most abundant living organisms on the planet, bacteria are outnumbered by a factor of 10 to 1 by phages which infect them [180]. As such, they have evolved various phage resistance mechanisms including preventing phage adsorption, preventing phage DNA entry, cutting of phage nucleic acids, and abortive infection systems [181]. Therefore, the use of phage cocktails can aid in overcoming limitations associated with the use of monospecific bacteriophages, such as phage resistance development and limited host range profile [61,182]. Phage cocktails broaden the phage host range and improve treatment efficacy by increasing the number of targeted pathogens. Different phages in cocktails can also synergize by targeting different receptors on bacterial surfaces [183]. Phage cocktails have also been reported to be economically advantageous in comparison to the “personalized medicine” approach of other phage therapy treatments [183]. Improved bioengineering capabilities may also benefit the use of phage cocktails through expanded host ranges and greater utility and specificity in combination therapies with antibiotics, for example [61]. Moreover, the stability of selected phages under a variety of storage and application stages must be considered in order to ensure their durability within intended-use environments [67]. Numerous external factors may influence the integrity of bacteriophages and must be carefully considered when preparing phage preparations for therapeutic and biocontrol use, including temperature, acidity, and salinity or ion concentration [184]. Crucially, the varying levels of these parameters under physiological conditions (e.g., low stomach pH) or industrial settings (e.g., variable temperature) offers a challenge when selecting appropriate phages. As such, efforts to preserve, prolong, and optimize selected phages against the influence of these external factors include the utilization of adapted phage evolution, as well as phage formulation, stabilization, and encapsulation techniques [185,186,187,188]. For example, Kering et al. 2020 were able to induce improved thermal stability of phages at elevated temperatures without affecting their lytic activity [189]. Effective methods for the rapid enhancement of additional, desired characteristics among selected phages continue to be developed as well [190].

### 5.2. Advantages of Bacteriophages as Biocontrol and Therapeutic Tools

The advantages which lytic bacteriophages provide for biocontrol and therapeutic purposes over traditional chemical antibacterial agents can be attributed to the very properties of phages. They may be applied across a variety of fields, most notable of them agriculture, food processing, and medicine. As bacterial viruses, their potential infection of mammalian cells is unlikely. Available evidence indicates that they have low inherent toxicity [3]. Toxicity tests have shown that no abnormal histological changes or impacts on morbidity or mortality were observed on tested rats who received phage doses orally [191]. While little evidence exists displaying any inherent toxicity associated with phage virions and potential anaphylactic immune responses, toxicity associated with impurities in phage preparations can pose an issue, although this can be minimized through proper phage purification [192,193]. In humans, treatment of various infections with phage administered via oral, superficial, intramuscular, or intravenous routes have been reported in different countries, and any apparent side effects were often minor or localized in nature [4,194,195]. Another benefit to the use of bacteriophages is that they are naturally sourced and highly abundant in a variety of environmental locations [3]. They can be viewed as “green”, and their environmental impact is minimal in comparison to standard chemical antibacterial agents [196]. Their natural abundance means that they are also easily discoverable, particularly in environments with high bacterial concentrations where phages can be isolated against most target bacteria [77]. They are commonly isolated from soil, water, food, sewage and waste management environments, with reports of aquatic environments containing 10^9^ phages/mL [77]. Bacteriophages have also been isolated from a variety of foods such as fresh chicken, beef, pork, and numerous raw vegetables, among others [197]. Moreover, bacteriophages exhibit a great deal of specificity in their targeted inactivation of a narrow spectrum of pathogenic bacteria [4]. Due to their intrinsic host specificity, phages can have the ability to infect only a handful of bacterial strains, or have the capacity to infect bacteria across more than one relatively closely related genus [3]. A major benefit to their host specificity is that phages have minimal impact on beneficial commensal bacteria found in the surrounding flora in contrast to the substantial effects which broad spectrum antibiotics can have on these bacterial populations [3]. Additionally, the continuous propagation of bacteriophages in the presence of their metabolically active host cells can allow for them to be used as a form of “active” therapy, as their antibacterial effects can be amplified through autodosing [3]. The mechanisms by which phages infect and inactivate bacteria differ from those of antibiotics, such that specific antibiotic resistance mechanisms generally do not confer cross-resistance to mechanisms of phage resistance [1]. Furthermore, bacteriophages exhibit relatively high versatility and stability in storage [67] and can be administered in a variety of forms such as liquids, creams, or impregnated solids [198,199], along with their previously mentioned suitability for diverse administration routes. The myriad of properties which make bacteriophages so versatile is a major pushing force for their development and application across numerous industries.

### 5.3. Bacteriophage as a Biocontrol Tool to Enhance Food Safety

Bacteriophages have received considerable attention in regard to their potential role in improving food safety throughout the food production and processing chain. Bacteriophage biocontrol can be utilized in pre-harvest and post-harvest scenarios, as well as in the decontamination of contact surfaces [200]. Bacteriophages may be administered to live animals via animal feed or spray-applied to feathers or hides prior to slaughter, or as phage preparations upon slaughter which are directly applied to food surfaces via direct spraying, though packaging materials, and as surface disinfectants in food processing plants [200]. The use of bacteriophages for biocontrol poses an attractive alternative for the sanitization of ready-to-eat foods such as fruits, vegetables, and meat products as a means to mitigate the chances of illness outbreaks following human consumption [201]. Common foodborne pathogens such as *Listeria* spp., *Salmonella* spp., *Campylobacter* spp., and *E. coli* have been the primary targets of phage biocontrol preparations due to the significant number of food-borne outbreaks associated with them [29]. For example, the European Union recorded that 21.8% of all outbreaks in 2015 were caused by *Salmonella* spp., and 8.9% of outbreaks were caused by *Campylobacter* spp. [202]. An average of 6500 cases of salmonellosis were also reported annually in Canada between 2009 and 2013 [203], while *Salmonella* spp. was noted to be the causative pathogen for 34% of confirmed single-pathogen illnesses and 66% of confirmed single-pathogen, outbreak-related hospitalizations in the United States in 2017 [204]. Additionally, over 265,000 cases of food illness related to Shiga toxin-producing *E. coli* (STEC) are estimated annually in the United States as well [205]. 

*Listeria* phages such as P100 and A511 have been shown to be effective in controlling *Listeria monocytogenes* counts in soft cheeses, mozzarella cheese brines, and chocolate milk [191,206]. Commercial phage biocontrol preparations such as ListShield^TM^ and Listex^TM^ have also been shown to reduce or eliminate *L. monocytogenes* contamination in a variety of fruits and vegetables, smoked fish, and sliced meats [200]. ListShield^TM^ has been shown to reduce *L. monocytogenes* counts by approximately 2.2 logs in prepackaged frozen foods [207]. Conversely, Listex^TM^ was shown to reduce *L. monocytogenes* levels by 2.8 logs to undetectable levels in ham slices [208]. Additionally, a separate study found that addition of Listex P100 phage preparations resulted in the 10-fold reduction of *L. monocytogenes* counts in tested ham samples as well [209]. Reductions in *Salmonella enterica* counts have been demonstrated upon the application of the commercial six-phage cocktail SalmoFresh^TM^, with average reductions of 5 logs on lettuce and 0.8 log on sprouts observed [210]. SalmoFresh^TM^ has been shown to be effective in reducing *Salmonella* amounts in poultry, where reductions of 1.2 logs in chicken breast and 1.3 logs in turkey breast have been observed [211,212]. While studies examining the use and efficacy of bacteriophages in reducing contamination of various foods by *Campylobacter* are limited, a study found a 0.7 log reduction of *Campylobacter* presence on chicken neck skins upon the use of phages [213]. Another group observed 1–3 logs reduction in *Campylobacter* counts on artificially contaminated raw and cooked beef slices [214]. Moreover, investigators have found a three-phage preparation which enables the near or complete elimination of *E. coli* O157:H7 from 78% of contaminated beef samples [215]. A separate three-phage cocktail was used in ground beef samples and was found to reduce *E. coli* O157:H7 levels by 1.2 logs [216]. The preservative effects of phages in raw chilled beef have also been recorded, with the shelf life of phage-treated beef being significantly extended [217]. Furthermore, the effectiveness of phage biocontrol on fresh produce has been demonstrated through the observed reductions of *E. coli* O157:H7 counts by 1–4 logs on baby spinach and green peppers, and reductions of *E. coli* O157:H7 counts by 2 logs on lettuce and 2–3 logs on cantaloupe as well [218]. Phage cocktails have also been shown to be effective in reducing *Listeria* presence by 2–4.6 logs in honeydew melons [219,220]. Phage biocontrol has also been employed in controlling plant diseases such as bacterial spot, bacterial wilt, and fire blight in potatoes, tomatoes, and mushrooms, among other cultivated plants [221]. For example, lytic bacteriophages against *Erwinia amylovora* have been isolated and successfully applied to control fire blight disease in apple and pear trees [222,223,224,225]. Black rot in broccoli caused by *Xanthomonas campestris* pv. *campestris* was significantly reduced upon treatment with bacteriophage as well [226]. Additionally, phage cocktails have been employed against *Ralstonia solanacearum* in bananas [227]. Several recent reviews have covered advancements and future prospective of using bacteriophages for the management of bacterial plant diseases [221,228,229,230,231]. In addition, the feasibility of bacteriophages to be used in aquaculture settings continues to be increasingly investigated, in both prophylactic and therapeutic approaches [232,233,234].

### 5.4. Use of Bacteriophages Against Biofilms

Bacteriophages have also been proposed as biocontrol agents for the decontamination of contact surfaces, with particular emphasis on their role in controlling the formation of biofilms by pathogenic bacteria. Biofilms have been shown to allow pathogens to persist in environments such as those in food processing and medical environments for prolonged periods, while resisting treatment with traditional antimicrobial and sanitizing agents [67,235]. Observations of a bacteriophage’s ability to penetrate biofilms, in addition to reported phage-antibiotic synergy, points to the potential use of bacteriophages as substitutes or supplements for biofilm elimination [236,237]. The use of single phage preparations and phage cocktails has been tested against biofilms [237], and phage cocktails have been observed to aid in preventing biofilm formation and improving biofilm eradication [238]. Specifically, the success of phage cocktails can be attributed to the difficulty they pose to bacteria in developing phage resistance, given that phage-resistant variants of *Pseudomonas aeruginosa* have been shown to develop within 6 h following infection with single phage preparations [239]. Phage cocktails have proven effective in reducing the colonization of *P. mirabilis* in dynamic biofilm model simulations [240]. Similar studies have shown the success of three-phage cocktails in eliminating *P. mirabilis*, which causes catheter-associated urinary tract infections [241]. Further, the reduction of *Staphylococcus aureus* counts in biofilms on titanium surfaces shows to be an important development in orthopedic implant-associated infections [242]. Moreover, seven log reductions of oral infection-associated *Enterococcus faecalis* have been observed within 48 h following phage treatment [243]. In addition to the use of whole phages for the reduction of bacterial biofilms, specific phage-encoded enzymes called depolymerases have been investigated for their ability to degrade the biofilm EPS [244]. These diverse phage-derived enzymes offer another avenue in which biofilm eradication can be achieved through the use of phage-based therapy [245,246]. The application of bacteriophages in clinically relevant biofilms associated with orthopedic, oral, and urinary tract infections has also been extensively covered recently [237]. Moreover, the application of bacteriophages to reduce *Listeria* biofilm formation on stainless steel surfaces simulating those found often in food-processing plants has also been reviewed [247]. Regulatory and safety considerations have also been discussed in regard to the use of whole phages and phage-based proteins for the control of biofilms within the food industry [248]. Thus, bacteriophages have been shown to be extremely versatile in their use against a variety of foodborne pathogens and biofilm-forming bacteria, warranting further consideration towards their research and regulatory approval for use in the agriculture and food industries.

### 5.5. Therapeutic Use of Bacteriophages

One of the most exciting opportunities for the application of bacteriophages is the use of phage therapy for clinical and therapeutic purposes. The activity of lytic bacteriophages against drug-resistant bacteria provides one of the areas where phage therapy can have the greatest impact in clinical use. In particular, their potential use in combatting the 12 Priority Pathogens identified by the World Health Organization is of particular interest (Table 2) [249]. As discussed above, findings have displayed a lack of adverse effects on patients following treatment with bacteriophages. The first US-FDA approved Phase I clinical trial for phage therapy, conducted in 2008, found no safety issues associated with the use of phage-based cocktails for the treatment of *E. coli*, *P. aeruginosa*, and *S. aureus* infection in patients suffering from chronic venous leg ulcers [250]. Additionally, *P. aeruginosa* colony counts have been observed to decrease following the application of six-phage cocktail preparations in the ears of patients suffering from chronic bacterial otitis, while patients and physicians reported decreased intensity of symptoms and no adverse effects [251]. Human clinical trials have also shown that the use of phage therapy in severe *Staphylococcus aureus* infections resulted in clinical improvements in 8 of 13 (62%) of patients, with no adverse reactions being reported as well [252]. The use of PhagoBioDerm, a commercially available phage-based antimicrobial wound dressing, also pointed to rapid clinical improvements in patients suffering from antibiotic resistant *S. aureus* infections within the course of a week [253]. Phage therapy has also been used in the United States to successfully treat a patient with an *A. baumannii* pancreatic pseudocyst infection [254]. Recent trials involving mice have shown a 2.3-fold increase in survival following the treatment of multidrug-resistant *A. baumannii* infection with bacteriophage cocktails [255]. Benefits have also been observed regarding the action of bacteriophages against lung infections caused by *Pseudomonas aeruginosa*, with encouraging effects on the host’s immune response being observed as well [256]. The “immunophage synergy” exhibited between the host immune system and bacteriophages provides another avenue for the success of clinical phage therapy, as their ability to aid the immune system while being tolerated by surrounding lung tissue may prove to be of benefit in combatting acute respiratory pathogens [256]. Moreover, the topical application of bacteriophages has been shown to be effective in aiding the recovery of patients suffering from *S. aureus*-associated antibiotic-unresponsive diabetic foot ulcers [257]. Previous reviews have covered the compassionate use of bacteriophage therapy for acute or chronic infections in humans, particularly within the 21st century, more extensively [258].

Finally, the potential for combined therapies against bacterial infections has been investigated, particularly the simultaneous use of bacteriophages with traditional antibiotics. One of the key advantages which this provides is that as these agents have distinct modes of action, there is a limited chance for the direct development of cross-resistance [259], although some findings have suggested that phage resistance mechanisms may pleiotropically confer increases in antibiotic resistance [260]. Another benefit to combination therapy utilizing phages and antibiotics is that phage production may be increased in the presence of sublethal concentrations of certain antibiotics [261], enabling enhanced bacterial suppression and the more efficient penetration and utility of the agents used [262], potentially aiding in decreasing the amount of antibiotics consumed. Examination of the phage–antibiotic combination has resulted in observations pointing towards potential synergistic effects, termed phage–antibiotic synergy (PAS). Synergistic effects have been observed by [263] upon the use of phage ECA2 and the antibiotic ciprofloxacin against the *E. coli* strain ATCC 13706. Furthermore, the use of phage SAP-26 in conjunction with rifampicin against the *Staphylococcus aureus* clinical isolate D43-a resulted in the death of 65% of present bacteria, while phage combinations with vancomycin and azithromycin yielded bacterial death rates of 40% and 60%, respectively [264]. The application of phage LUZ7 in combination with streptomycin against *Pseudomonas aeruginosa* resulted in reductions in bacterial cell densities significantly greater than each individual treatment [265]. Interestingly, findings have also displayed that mutations associated with developing phage resistance such as *sag*A, *epa*R, and *epa*X may enhance the susceptibility of *Enterococcus faecium* to ceftriaxone, an antibiotic which is normally ineffective against *E. faecium* [266]. Nonetheless, further considerations are needed regarding the combined use of bacteriophages and antibiotics against bacterial infections regarding the choice of phage and antibiotic, and the ratios of each agent used [262].

**Table 2:** Examples of published studies employing bacteriophage therapy with success against bacterial pathogens identified as part of the World Health Organization’s Priority Pathogens since 2010.

Bacterial Pathogen	Phage	Subject/Model	Details	Reference
*Acinetobacter baumannii*	Βϕ-R2096	*Galleria mellonella* larvae Mouse	Increased survival rates in both larvae and mice models. No mortality or serious side effects observed in phage-treated groups.	[267]
*Acinetobacter baumannii*	Phage Cocktail (5 phages)	Human patient	Intravenous treatment Slight improvements in alertness, no signs of further infection. Patient died after decision to withdraw care by family.	[268]
*Acinetobacter baumannii*	PBAB08 PBAB25	Mouse	Intraperitoneal, intranasal, and oral treatment. 2.3-fold higher survival rate than untreated subjects within 7 days. None or minimal inflammatory responses recorded.	[255]
*Pseudomonas aeruginosa*	Phage Cocktail (4 phages)	Zebrafish	Decreased lethality, bacterial burden, and pro-inflammatory response caused by bacterial infection.	[269]
*Pseudomonas aeruginosa*	BrSP1	In vitro	Maintenance of bacterial population at low levels 12 h post infection. Host range analysis exhibits 51.4% of 26 investigated bacterial strains were susceptible.	[270]
*Pseudomonas aeruginosa*	MAG1 MAG4	In vitro	MAG4 reduced present biofilm formations more effectively after short treatment time. MAG1 was more effective with longer treatment time and selected less for phage-resistant clones.	[271]
*Staphylococcus aureus*	STA1.ST29 EB1.ST11 EB1.ST27	In vitro	Phage cocktail was able to reduce bacterial germ density in pasteurized milk and raw milk. Only moderate decreases in raw milk results compared to pasteurized milk observed.	[272]
*Staphylococcus aureus*	AB-SA01	Human patients	Intravenous administration. 8 of 13 patients showed signs of clinical improvement, while no adverse reactions were reported or attributed to the application of phages.	[252]
*Escherichia coli*	Phage Cocktail (ListShield^TM^, EcoShield PX^TM^, SalmoFresh^TM^)	Mouse	Phage cocktail significantly reduced bacterial pathogen counts by 54% in fecal samples. No notable changes or distortion of gut microbiota composition. Decreased weight-loss occurred in mice treated with phage cocktail compared to other treatment groups.	[273]
*Escherichia coli*	Phage Cocktail (ECML-363, ECML-122, ECML-359)	In vitro	Phage cocktail more effective than ciprofloxacin administration in reducing simulated bacterial populations (2–3 log reduction). No to moderate impact on commensal bacteria observed compared to antibiotic.	[274]
*Escherichia coli*	CS EPEC BL EHEC	In vitro	High efficiency in reduction of EPEC or EHEC contaminated meat, in about 99.20% and 99.04% respectively.	[275]
*Salmonella* spp.	LPSTLL LPST94 LPST153	In vitro	Phage cocktail had broad spectrum to lyse diverse *Salmonella* serovars. Near complete elimination of targeted pathogens in milk samples after 6 h and 12 h of phage treatment.	[276]
*Salmonella* spp.	Phage Cocktail (5 phages)	In vitro	Reductions of 1.0 log CFU/cm^2^ observed following immersion of samples (chicken skins) in phage suspensions.	[277]
*Campylobacter* spp.	Phage Cocktail	Broiler chicken	Significant reduction and control of C. jejuni presence within 24 h of phage application. Continued presence of phages 6 days after phage application.	[278]

## 6. Bacteriophages for Detection of Bacterial Pathogens

In addition to their use as therapeutic agents against bacterial pathogens, bacteriophages also have the potential to be used for the rapid, specific, and sensitive detection of bacterial pathogens [279]. Their innate receptor specificity allows for the development of assays tailored to capture target bacteria, and their abundance in nature and ability to propagate within host cells provides increased sensitivity for assays using the “built-in” amplification system while also making them inexpensive and easy to produce [67]. While traditional culture-based detection remains the gold-standard for pathogen detection, these techniques are labor- and time-intensive, requiring 3–5 days for accurate results to be obtained [280]. Alternatively, bacteriophage-based detection allows for the more rapid detection of pathogens as the entire infection process only takes 1–2 h [67]. 

While the notion of using bacteriophages for the detection of bacterial pathogens has been examined throughout the past several decades, few examples of commercially available phage-based diagnostic tests have materialized. This can be attributed to the lack of sufficient knowledge on phage biology and genetic structure [67], in addition to the lack of protocols which ensure proper sensitivity, stability, and reproducibility required for phage-based detections methods to be successful [281]. Nonetheless, recent developments and extensive research have the potential to yield significant advantages for the improved sensitivity, specificity, and rapidity of phage-based detection methods [282]; paving the path for the increased impact of these methods and a move away from culture-based detection techniques [283]. Phage-based detection methods have been characterized in relation to their mechanism of action, with infection-based and capture-based detection methods being most prevalent [281], as briefly highlighted below.

### 6.1. Infection-Based Detection

Infection-based detection utilizes the phage genome integration step during the lysogenic phage life cycle and rapid progression of the lytic cycle with the release of phage progeny or bacterial cell contents such as DNA, RNA, or bacterial proteins to be used as markers [281]. Lytic and lysogenic phages can be engineered to encode reporting elements to be used as markers, as is the case with luminescent, fluorescent, and colorimetric detection assays [284]. Upon host infection, the expression of reporter proteins such as fluorescent proteins, luciferases, and hydrolyzing enzymes aids in amplifying the detection signal with the addition of a substrate [285]. Reporter phages which have been engineered to exhibit bioluminescence upon infection of targeted bacterial hosts have been used for the detection of *Staphylococcus aureus* [286], *Listeria monocytogenes* [287], *Salmonella* spp. [288], and *E. coli* O157:H7 [289], among other bacterial pathogens [67]. Additionally, bacteriophages encoding green fluorescent protein have been used to detect both *E. coli* and *Salmonella* within the span of 1 h [290]. However, luminescent detection has been noted as being the most sensitive method of detection due to the lack of background bioluminescence within the majority of samples, in comparison to fluorescent detection which could potentially be inhibited by stronger autofluorescence signals originating from a sample [67]. In addition to the detection of targeted bacteria following phage-mediated infection and lysis, the inhibition or delay in bacterial growth may also be used for detection purposes [279]. By monitoring for changes or delays in the electrical properties of growth media, the presence of bacterial species of interest in light of the presence of infecting phages may be detected [279]. This was accomplished by Chang et al. 2002 through their identification of *E. coli* O157:H7 via incorporation of conductimetric measurement and phage AR1, yielding near 100% sensitivity and specificity [291]. Additionally, protocols for the combined use of bacteriophage D29; as capturing and lysing agent; and PCR for amplification of DNA have enabled the rapid and sensitive detection and identification of viable *Mycobacterium paratuberculosis* from clinical blood samples within 6 h, with a limit of detection of ≤10 cells/mL [292].

### 6.2. Capture-Based Detection

Capture-based detection employs the unique specificity of bacteriophages to utilize them as biosensors [281]. Capture-based techniques may utilize whole-phage particles or specific phage receptors as tag molecules, using their innate affinity to detect targeted pathogens without the need for phage infection [282]. The immobilization of phage virions enables them to be used as specific bioreceptors upon the detection of their binding to specific bacterial hosts [279,281]. One of the most common methods employed to detect the binding of whole phage particles is surface plasmon resonance, which has been used to detect methicillin-resistant *S. aureus* (MRSA) at levels of 10^3^ CFU/mL while also being able to distinguish methicillin-sensitive *S. aureus* from MRSA [293]. Conversely, the use of phage components as opposed to whole-phage particles offers a variety of benefits, including enhanced binding activity due to smaller probe sizes, improved specificity and affinity through engineering, and heightened robustness [279]. Specialized receptor binding proteins (RBPs) from tail fibers and spikes have been used in the glycotyping and identification of *Salmonella* strains [294] and *Listeria* strains [295]. In addition, genetically engineered tail-spike proteins from *Salmonella* phage P22 were used to create a biosensor that was able to detect real-time interactions of *Salmonella* cells at concentrations of 10^3^ CFU/mL [296]. Receptor binding proteins have also been used in the detection of *Campylobacter jejuni* and *Campylobacter coli*, with produced assays exhibiting 100% specificity to both pathogens, and 95% sensitivity for *C. jejuni* and 90% sensitivity for *C. coli* [297]. Moreover, labelled cell wall binding domains (CBDs), sourced from phage endolysin enzymes, have also been used and proven effective in the capture and detection of various Gram-positive bacterial pathogens such as *Listeria monocytogenes*, *Bacillus cereus*, and *Clostridium perfringens* [282]. They have exhibited remarkable versatility, with their binding spectra being observed to be broader than the host ranges of corresponding phages [298], to displaying specificity at the serovar or strain level in the case of studied *Listeria* CBDs [299,300].

## 7. Future Perspectives and Applications of Bacteriophages in the Fight against Antimicrobial Resistance

While phage therapy suffered from a lack of support throughout the late 20th century due to the insufficient understanding of bacteriophage biology, current hurdles are primarily limited by knowledge of industrial applications and associated limits in regulatory approval and availability [301]. With phage research continuing to focus on interactions among infecting bacteriophages and their bacterial hosts, artificially inoculated samples and experimental conditions in pre-clinical and biocontrol studies may lack the ability to reflect real-world scenarios [67,302]. Thus, the examination of the safety and efficacy of bacteriophages in the form of clinical trials, field trials, and phage–host interaction studies is urgently needed in order for phage therapy to be considered as a viable tool to combat antibacterial resistance. As studies have previously considered the importance of phage–host interactions [303] and the topic has received more attention recently [304,305,306], the growing understanding of underlying bacteriophage mechanisms may provide key insight into how they may be applied in more complex environments. Moreover, advances in genome sequencing and bioinformatic prediction programs have enabled phages to be more thoroughly screened to minimize drawbacks associated with the transduction of undesired factors [177]. While phages have traditionally been screened in terms of plaque morphology, this may lead to non-definitive results in relation to their reproductive cycle [177]. As such, the use of genome sequencing in recent years has increased as a result of cost-effective ability to simultaneously screen for multiple properties such as integration/excision genes, toxin/resistance genes, and transduction potential [307,308]. This will enable for therapeutic phages to be screened more effectively, aiding both their utility and minimizing associated costs. 

Improvements in computational biology and sequencing technologies have also furthered understanding in relation to the diversity and complexity of the bacteriophage community (phageome). While phages have been found to be ubiquitous in a variety of natural environments, with estimates of their presence in the range of 10^31^ viral particles across the biosphere [65], the form and function of the collective phageome as part of the human gut microbiome has been of particular interest recently. Estimates have shown that nearly 10^8^–10^10^ viral particles may exist per gram of human feces, and of phages outnumbering bacteria by a factor of 20 to 1 [309]. As the viral component of the human microbiome is presumed to be dominated by bacteriophages [310], temperate phages are believed to play a key role in shaping the bacterial community through horizontal gene transfer [311]. The sheer presence and diversity of available bacteriophages has allowed for disease-specific alterations to the phageome to be observed in relation to a number of gastrointestinal and systemic disorders, including inflammatory bowel disease [312], AIDS [313], malnutrition [314], and childhood obesity [315]. Nonetheless, the human gut phageome remains an area with plenty of “dark matter” yet to be recognized in comparison to the better understood phage marine ecosystems [309]. The growing body of knowledge regarding the human gut phageome will undoubtedly ignite more phageome studies of food production and processing environments and apply this information to improve food safety spanning the “farm to fork” model. 

As research involving bacteriophages has progressed, interest regarding the underlying intricacies of the bacterial viruses has grown, including the utilization of specific phage components or mechanisms, as well as exploiting potential crossover with emerging synthetic biology practices. The ability to create/adapt specialized phages through novel phage engineering techniques allows for various improvements on existing phages to be carried through, including host range expansion and improving phage antibacterial properties [316]. Tail fiber proteins and adsorption structures have been the target of modifications as a means to overcome some of the limitations associated with the intrinsically narrow host ranges of bacteriophages [316]. For example, the identification of a hybrid T3/T7 phage displaying improved adsorption efficiency compared to both individual T3 and T7 phages provides promising prospect in regard to exploiting the natural diversity of phages [317]. In addition, Dunne et al. 2019 were able to extend the host range of *Listeria* phage PSA to target a variety of *Listeria* serovars through the alteration of receptor-binding proteins (RBP), from targeting only the SV 4b RBP domain to targeting SVs 4a, 4b, 4d, and 5 [318]. Moreover, specific phage-derived proteins have been investigated as possible alternatives to whole phage applications, aiding to overcome some of their associated disadvantages such as the previously discussed phage resistance development. Phage endolysins have been one of the most examined phage-encoded enzymes, owing to their essential role in mediating the release of phage progeny during the final stages of the phage life cycle, which in turn may be exploited to aid in the lysis and death of selected bacteria [319,320]. For example, endolysins such as LysIME-EF1 have exhibited efficient antibacterial activity against multiple strains of *Enterococcus faecalis* [321], while the endolysin Ts2631 of phage vB_Tsc2631 has been proposed as a model for future antimicrobial agents due to its intrinsic bactericidal activity and uncommon thermal stability [322]. Additionally, endolysin LysMK34 of phage PMK34 has exhibited intrinsic antibacterial activity up to 2.4 and 4.8 log reductions in *Pseudomonas aeruginosa* and *Acinetobacter baumannii*, as well as aiding to resensitize colistin-resistant bacterial strains [323]. The application of endolysins has focused on their utility against Gram-positive bacteria due to the lack of an outer membrane barrier which can prevent access to the peptidoglycan, which has thus limited their use against Gram-negative counterparts [324]. Thus, Briers et al. 2014 sought to fuse lipopolysaccharide (LPS)-destabilizing proteins to endolysin PVP-SE1gp146 to generate Artilysins, which harness various physicochemical properties (hydrophobic, amphipathic, or cationic) to act as highly effective outer-membrane penetrating bactericidal agents (4–5 log reductions) against common Gram-negative pathogens such as *Pseudomonas aeruginosa* and *Acinetobacter baumannii* [325]. Additionally, Zampara et al. 2018 engineered innolysins by combining receptor binding proteins (RBPs) with candidate phages, such as RBP Pb5 with phage T5, which were able to overcome the outer membrane barrier and led to observed inhibitory effects on *E. coli* ATCC11303 and reductions in the number of bacteria by 1 log [326]. In addition to phage enzyme-based antimicrobials and phage therapy, advancements in genome-driven screening, annotation, and interaction modelling have improved our understanding of hypothetical proteins with once unknown function. These small and early genes have been targeted for their potential as novel polypeptides with bactericidal properties [327]. Phage-based hijacking proteins and mechanisms may be suitable in either uncovering new targets for existing antibacterial agents or for novel antimicrobials altogether [327]. Furthermore, while current studies exploring phage-based therapeutics may focus on a traditional approach in directly killing bacterial cells or inhibiting their growth, new phage-based antivirulence strategies may aim to disarm bacterial pathogens and render them less virulent or more susceptible to other avenues of removal [244]. For example, while phage cocktails, phage engineering, and combination therapies involving phages and antibiotics may be used to reduce occurrences of phage resistance; an evolutionary trade-off may be harnessed by using phage resistance itself as part of the therapeutic strategy to increase the sensitivity of targeted bacteria to antimicrobials or the immune system [244]. 

## 8. Conclusions

In this review, we discussed the role which bacteriophages play in the horizontal gene transfer of genes associated with antimicrobial resistance and virulence, in addition to some of the applications and uses which bacteriophages are being studied for. The diversity and innate specificity of whole phage particles and phage components, coupled with the enormous presence of phages within different environments [77], means that bacteriophages have great potential to be considered as emerging tools in therapy, biocontrol, and detection. Improved understanding of their remarkable specificity can also enable greater care in selecting correct bacteriophages for use during critical moments, in which they can have the most positive effect. Phage research has attracted considerable attention recently to alleviate impacts associated with the emergence of antimicrobial resistance in pathogenic bacteria, and the regulatory approval and commercialization of several phage-based products is a positive sign for this rapidly evolving field [249,328,329,330].

Considerable understanding of the mechanisms and genetics behind bacteriophage transduction is needed in order to reduce its impacts and can lead to greater interpretation of its roles and implications both within the natural environment and on its potential in regard to therapeutic phages. Advances in genome sequencing technologies and metagenomics, including the emergence of the field of transductomics [331], have the potential to aid in the proactive prediction of gene transfer potential and of improving our understanding surrounding viral dark matter. Meanwhile, improvements on existing bacteriophage isolation, characterization, preparation, and delivery protocols can ensure that such antibacterial agents are safe and effective, with minimal transduction potential. Furthermore, continued examination of phage–host interactions can aid in overcoming associated challenges with phage-based biocontrol and therapy and lead to the optimization of such treatments. The roles and impact of phages on bacterial hosts and the microbial community to which they are introduced are yet to be fully understood and should be an important prerequisite to their wide-scale acceptance and implementation.

Currently, research involving bacteriophages seems to be mostly restricted to academic environments, with interest appearing to be lacking across industrial partners amid fears of steep costs over profitability [332]. While bacteriophages are not the magic bullet to be considered as complete alternatives or replacements of traditional antibacterial agents such as antibiotics, promising results continue to be found in regard to combination therapies and as additions to the hurdle technology system throughout the food production and processing chain. However, as our understanding of the ubiquitous bacterial viruses expands, their role continues to evolve, and our appreciation for these ancient organisms and their storied histories grows, from their initial examination as research organisms to potentially necessary agents in the fight against rising antimicrobial resistance. Therefore, the most exciting aspect of bacteriophages and their numerous applications and potential benefits lies not within their storied past, but within the boundless future they offer.

## References

[1] Moghadam M.T., Amirmozafari N., Shariati A., Hallajzadeh M., Mirkalantari S., Khoshbayan A., Masjedian Jazi F. (2020). How Phages Overcome the Challenges of Drug Resistant Bacteria in Clinical Infections. Infect. Drug Resist..

[2] Lin D.M., Koskella B., Lin H.C. (2017). Phage therapy: An alternative to antibiotics in the age of multi-drug resistance. World J. Gastrointest. Pharmacol. Ther..

[3] Loc-Carrillo C., Abedon S.T. (2011). Pros and cons of phage therapy. Bacteriophage.

[4] Abedon S.T., Kuhl S.J., Blasdel B.G., Kutter E.M. (2011). Phage treatment of human infections. Bacteriophage.

[5] Fernández L., Gutiérrez D., Rodríguez A., García P. (2018). Application of Bacteriophages in the Agro-Food Sector: A Long Way Toward Approval. Front. Cell. Infect. Microbiol..

[6] Reuter M., Kruger D.H. (2020). Approaches to optimize therapeutic bacteriophage and bacteriophage-derived products to combat bacterial infections. Virus Genes.

[7] Cook M., Molto E., Anderson C. (1989). Fluorochrome labelling in roman period skeletons from dakhleh oasis, Egypt. Am. J. Phys. Anthropol..

[8] Nelson M.L., Dinardo A., Hochberg J., Armelagos G.J. (2010). Brief communication: Mass spectroscopic characterization of tetracycline in the skeletal remains of an ancient population from Sudanese Nubia 350–550 CE. Am. J. Phys. Anthropol..

[9] Aminov R.I. (2010). A brief history of the antibiotic era: Lessons learned and challenges for the future. Front. Microbiol..

[10] Luepke K.H., Suda K.J., Boucher H., Russo R.L., Bonney M.W., Hunt T.D., Mohr J.F. (2017). Past, Present, and Future of Antibacterial Economics: Increasing Bacterial Resistance, Limited Antibiotic Pipeline, and Societal Implications. Pharmacotherapy.

[11] Golparian D., Harris S.R., Sánchez-Busó L., Hoffmann S., Shafer W.M., Bentley S.D., Jensen J.S., Unemo M. (2020). Genomic evolution of Neisseria gonorrhoeae since the preantibiotic era (1928–2013): Antimicrobial use/misuse selects for resistance and drives evolution. BMC Genom..

[12] Gottfried J. (2005). History Repeating? Avoiding a Return to the Pre-Antibiotic Age. Digit. Access Scholarsh. Harv..

[13] Aminov R. (2017). History of antimicrobial drug discovery: Major classes and health impact. Biochem. Pharmacol..

[14] Christaki E., Marcou M., Tofarides A. (2020). Antimicrobial Resistance in Bacteria: Mechanisms, Evolution, and Persistence. J. Mol. Evol..

[15] Ventola C.L. (2015). The antibiotic resistance crisis: Part 1: Causes and threats. Perspect. Med. Chem..

[16] Fair R.J., Tor Y. (2014). Antibiotics and bacterial resistance in the 21st century. Perspect. Med. Chem..

[17] Michael C.A., Dominey-Howes D., Labbate M. (2014). The Antimicrobial Resistance Crisis: Causes, Consequences, and Management. Front. Public Health.

[18] Luyt C.-E., Bréchot N., Trouillet J.-L., Chastre J. (2014). Antibiotic stewardship in the intensive care unit. Crit. Care.

[19] Lushniak B.D. (2014). Antibiotic resistance: A public health crisis. Public Health Rep..

[20] Andersson D.I., Hughes D. (2014). Microbiological effects of sublethal levels of antibiotics. Nat. Rev. Microbiol..

[21] Llor C., Hernández S., Bayona C., Moragas A., Sierra N., Hernández M., Miravitlles M. (2013). A study of adherence to antibiotic treatment in ambulatory respiratory infections. Int. J. Infect. Dis..

[22] Llor C., Bjerrum L. (2014). Antimicrobial resistance: Risk associated with antibiotic overuse and initiatives to reduce the problem. Ther. Adv. Drug Saf..

[23] Maillard J.-Y., Bloomfield S.F., Courvalin P., Essack S.Y., Gandra S., Gerba C.P., Rubino J.R., Scott E.A. (2020). Reducing antibiotic prescribing and addressing the global problem of antibiotic resistance by targeted hygiene in the home and everyday life settings: A position paper. Am. J. Infect. Control..

[24] Larsson D.G.J., Andremont A., Bengtsson-Palme J., Brandt K.K., de Roda Husman A.M., Fagerstedt P., Fick J., Flach C.-F., Gaze W.H., Kuroda M. (2018). Critical knowledge gaps and research needs related to the environmental dimensions of antibiotic resistance. Environ. Int..

[25] Bürgmann H., Frigon D., Gaze W.H., Manaia C.M., Pruden A., Singer A.C., Smets B.F., Zhang T. (2018). Water and sanitation: An essential battlefront in the war on antimicrobial resistance. FEMS Microbiol. Ecol..

[26] Landers T.F., Cohen B., Wittum T.E., Larson E.L. (2012). A review of antibiotic use in food animals: Perspective, policy, and potential. Public Health Rep..

[27] Diarra M.S., Malouin F. (2014). Antibiotics in Canadian poultry productions and anticipated alternatives. Front. Microbiol..

[28] Collignon P.J., McEwen S.A. (2019). One Health-Its Importance in Helping to Better Control Antimicrobial Resistance. Trop. Med. Infect. Dis..

[29] Atterbury R.J. (2009). Bacteriophage biocontrol in animals and meat products. Microb. Biotechnol..

[30] Economou V., Gousia P. (2015). Agriculture and food animals as a source of antimicrobial-resistant bacteria. Infect. Drug Resist..

[31] Kraemer S.A., Ramachandran A., Perron G.G. (2019). Antibiotic Pollution in the Environment: From Microbial Ecology to Public Policy. Microorganisms.

[32] Massé D.I., Saady N.M.C., Gilbert Y. (2014). Potential of Biological Processes to Eliminate Antibiotics in Livestock Manure: An Overview. Animals.

[33] Cycoń M., Mrozik A., Piotrowska-Seget Z. (2019). Antibiotics in the Soil Environment—Degradation and Their Impact on Microbial Activity and Diversity. Front. Microbiol..

[34] Davies R., Wales A. (2019). Antimicrobial Resistance on Farms: A Review Including Biosecurity and the Potential Role of Disinfectants in Resistance Selection. Compr. Rev. Food Sci. Food Saf..

[35] Argudín M.A., Deplano A., Meghraoui A., Dodémont M., Heinrichs A., Denis O., Nonhoff C., Roisin S. (2017). Bacteria from Animals as a Pool of Antimicrobial Resistance Genes. Antibiotics.

[36] Mcdonnell G., Russell A.D. (1999). Antiseptics and disinfectants: Activity, action, and resistance. Clin. Microbiol. Rev..

[37] Kapoor G., Saigal S., Elongavan A. (2017). Action and resistance mechanisms of antibiotics: A guide for clinicians. J. Anaesthesiol. Clin. Pharmacol..

[38] Sheldon A.T. (2005). Antiseptic “Resistance”: Real or Perceived Threat?. Clin. Infect. Dis..

[39] Carey D.E., McNamara P.J. (2015). The impact of triclosan on the spread of antibiotic resistance in the environment. Front. Microbiol..

[40] Yueh M.-F., Tukey R.H. (2016). Triclosan: A Widespread Environmental Toxicant with Many Biological Effects. Annu. Rev. Pharmacol. Toxicol..

[41] Condell O., Sheridan Á., Power K.A., Bonilla-Santiago R., Sergeant K., Renaut J., Burgess C., Fanning S., Nally J.E. (2012). Comparative proteomic analysis of Salmonella tolerance to the biocide active agent triclosan. J. Proteom..

[42] Thomas L., Maillard J.Y., Lambert R.J.W., Russell A.D. (2000). Development of resistance to chlorhexidine diacetate in Pseudomonas aeruginosa and the effect of a “residual” concentration. J. Hosp. Infect..

[43] Food and Drug Administration FDA Issues Final Rule on Safety and Effectiveness of Antibacterial Soaps. https://www.fda.gov/news-events/press-announcements/fda-issues-final-rule-safety-and-effectiveness-antibacterial-soaps.

[44] Barras F., Aussel L., Ezraty B. (2018). Silver and Antibiotic, New Facts to an Old Story. Antibiotics.

[45] Gerba C.P. (2015). Quaternary Ammonium Biocides: Efficacy in Application. Appl. Environ. Microbiol..

[46] Sundheim G., Langsrud S., Heir E., Holck A.L. (1998). Bacterial resistance to disinfectants containing quaternary ammonium compounds. Int. Biodeterior. Biodegrad..

[47] Reygaert W.C. (2018). An overview of the antimicrobial resistance mechanisms of bacteria. AIMS Microbiol..

[48] Munita J.M., Arias C.A. (2016). Mechanisms of Antibiotic Resistance. Microbiol. Spectr..

[49] Gyles C. (2011). The growing problem of antimicrobial resistance. Can. Vet. J. La Rev. Vet. Can..

[50] Dadgostar P. (2019). Antimicrobial Resistance: Implications and Costs. Infect. Drug Resist..

[51] Keen E.C. (2015). A century of phage research: Bacteriophages and the shaping of modern biology. Bioessays.

[52] Luria S.E., Delbrück M. (1943). Mutations of bacteria from virus sensitivity to virus resistance. Genetics.

[53] Hershey A.D., Chase M. (1952). Independent functions of viral protein and nucleic acid in growth of bacteriophage. J. Gen. Physiol..

[54] Zinder N.D., Lederberg J. (1952). Genetic exchange in Salmonella. J. Bacteriol..

[55] Crick F.H.C., Barnett L., Brenner S., Watts-Tobin R.J. (1961). General Nature of the Genetic Code for Proteins. Nature.

[56] Barrangou R., Marraffini L.A. (2014). CRISPR-Cas systems: Prokaryotes upgrade to adaptive immunity. Mol. Cell.

[57] Rath D., Amlinger L., Rath A., Lundgren M. (2015). The CRISPR-Cas immune system: Biology, mechanisms and applications. Biochimie.

[58] Ishino Y., Krupovic M., Forterre P. (2018). History of CRISPR-Cas from Encounter with a Mysterious Repeated Sequence to Genome Editing Technology. J. Bacteriol..

[59] Howard-Varona C., Lindback M.M., Bastien G.E., Solonenko N., Zayed A.A., Jang H., Andreopoulos B., Brewer H.M., Glavina del Rio T., Adkins J.N. (2020). Phage-specific metabolic reprogramming of virocells. ISME J..

[60] Warwick-Dugdale J., Buchholz H.H., Allen M.J., Temperton B. (2019). Host-hijacking and planktonic piracy: How phages command the microbial high seas. Virol. J..

[61] Altamirano F.L.G., Barr J.J. (2019). Phage Therapy in the Postantibiotic Era. Clin. Microbiol. Rev..

[62] Wittebole X., De Roock S., Opal S.M. (2014). A historical overview of bacteriophage therapy as an alternative to antibiotics for the treatment of bacterial pathogens. Virulence.

[63] Sulakvelidze A., Alavidze Z., Morris J.G. (2001). Bacteriophage therapy. Antimicrob. Agents Chemother..

[64] Myelnikov D. (2018). An Alternative Cure: The Adoption and Survival of Bacteriophage Therapy in the USSR, 1922–1955. J. Hist. Med. Allied Sci..

[65] Mushegian A.R. (2020). Are There 10^31 Virus Particles on Earth, or More, or Fewer?. J. Bacteriol..

[66] Ackermann H.-W. (2003). Bacteriophage observations and evolution. Res. Microbiol..

[67] Brovko L.Y., Anany H., Griffiths M.W., Henry J.B.T.-A. (2012). Chapter Six—Bacteriophages for Detection and Control of Bacterial Pathogens in Food and Food-Processing Environment.

[68] White H.E., Orlova E.V., Savva E.V.O.E.-R. (2019). Bacteriophages: Their Structural Organisation and Function.

[69] Callanan J., Stockdale S.R., Shkoporov A., Draper L.A., Ross R.P., Hill C. (2018). RNA Phage Biology in a Metagenomic Era. Viruses.

[70] Yuan Y., Gao M. (2017). Jumbo Bacteriophages: An Overview. Front. Microbiol..

[71] Devoto A.E., Santini J.M., Olm M.R., Anantharaman K., Munk P., Tung J., Archie E.A., Turnbaugh P.J., Seed K.D., Blekhman R. (2019). Megaphages infect Prevotella and variants are widespread in gut microbiomes. Nat. Microbiol..

[72] Ackermann H.-W., Prangishvili D. (2012). Prokaryote viruses studied by electron microscopy. Arch. Virol..

[73] Ackermann H.-W. (2007). 5500 Phages examined in the electron microscope. Arch. Virol..

[74] Principi N., Silvestri E., Esposito S. (2019). Advantages and Limitations of Bacteriophages for the Treatment of Bacterial Infections. Front. Pharmacol..

[75] Campbell A. (1961). Conditions for the Existence of Bacteriophage. Evolution.

[76] Żbikowska K., Michalczuk M., Dolka B. (2020). The Use of Bacteriophages in the Poultry Industry. Animals.

[77] Clokie M.R., Millard A.D., Letarov A.V., Heaphy S. (2011). Phages in nature. Bacteriophage.

[78] Kutter E., Sulakvelidze A. (2004). Bacteriophages: Biology and Applications.

[79] Dowah A.S.A., Clokie M.R.J. (2018). Review of the nature, diversity and structure of bacteriophage receptor binding proteins that target Gram-positive bacteria. Biophys. Rev..

[80] Lindberg A.A. (1973). Bacteriophage Receptors. Annu. Rev. Microbiol..

[81] Horáček P., Zárybnický V., Roubal J., Turková J., Dobišová M. (1970). Influence of NaCl, KCl and MgSO_4_ concentration on total and irreversible adsorption of T2r phage on isolated cell walls. Folia Microbiol. (Praha)..

[82] Fernandes S., São-José C. (2018). Enzymes and Mechanisms Employed by Tailed Bacteriophages to Breach the Bacterial Cell Barriers. Viruses.

[83] Click E.M., Webster R.E. (1997). Filamentous phage infection: Required interactions with the TolA protein. J. Bacteriol..

[84] Lipton A., Weissbach A. (1969). The bacteriophages. Am. J. Med..

[85] Dove W.F. (1966). Action of the lambda chromosome: I. Control of functions late in bacteriophage development. J. Mol. Biol..

[86] Aksyuk A.A., Rossmann M.G. (2011). Bacteriophage assembly. Viruses.

[87] Drulis-Kawa Z., Majkowska-Skrobek G., Maciejewska B., Delattre A.-S., Lavigne R. (2012). Learning from bacteriophages—Advantages and limitations of phage and phage-encoded protein applications. Curr. Protein Pept. Sci..

[88] Utter B., Deutsch D.R., Schuch R., Winer B.Y., Verratti K., Bishop-Lilly K., Sozhamannan S., Fischetti V.A. (2014). Beyond the chromosome: The prevalence of unique extra-chromosomal bacteriophages with integrated virulence genes in pathogenic Staphylococcus aureus. PLoS ONE.

[89] Howard-Varona C., Hargreaves K.R., Abedon S.T., Sullivan M.B. (2017). Lysogeny in nature: Mechanisms, impact and ecology of temperate phages. ISME J..

[90] Nanda A.M., Thormann K., Frunzke J. (2015). Impact of Spontaneous Prophage Induction on the Fitness of Bacterial Populations and Host-Microbe Interactions. J. Bacteriol..

[91] Gordo I., Perfeito L., Sousa A. (2012). Fitness Effects of Mutations in Bacteria. J. Mol. Microbiol. Biotechnol..

[92] Brüssow H., Canchaya C., Hardt W.-D. (2004). Phages and the Evolution of Bacterial Pathogens: From Genomic Rearrangements to Lysogenic Conversion. Microbiol. Mol. Biol. Rev..

[93] Pedulla M.L., Ford M.E., Houtz J.M., Karthikeyan T., Wadsworth C., Lewis J.A., Jacobs-Sera D., Falbo J., Gross J., Pannunzio N.R. (2003). Origins of Highly Mosaic Mycobacteriophage Genomes. Cell.

[94] Brown-Jaque M., Calero-Cáceres W., Muniesa M. (2015). Transfer of antibiotic-resistance genes via phage-related mobile elements. Plasmid.

[95] Huddleston J.R. (2014). Horizontal gene transfer in the human gastrointestinal tract: Potential spread of antibiotic resistance genes. Infect. Drug Resist..

[96] Valero-Rello A., López-Sanz M., Quevedo-Olmos A., Sorokin A., Ayora S. (2017). Molecular mechanisms that contribute to horizontal transfer of plasmids by the bacteriophage SPP1. Front. Microbiol..

[97] Thomas C.M., Nielsen K.M. (2005). Mechanisms of, and Barriers to, Horizontal Gene Transfer between Bacteria. Nat. Rev. Microbiol..

[98] Chiang Y.N., Penadés J.R., Chen J. (2019). Genetic transduction by phages and chromosomal islands: The new and noncanonical. PLoS Pathog..

[99] Bushman F. (2002). Lateral DNA Transfer.

[100] Touchon M., Bernheim A., Rocha E.P. (2016). Genetic and life-history traits associated with the distribution of prophages in bacteria. ISME J..

[101] Griffiths A.J.F., Miller J.H., Suzuki D.T., Lewontin R.C., Gelbart W.M., Freeman W.H. (2000). Introduction to Genetic Analysis.

[102] Schneider C.L. (2017). Bacteriophage-Mediated Horizontal Gene Transfer: Transduction.

[103] Matilla M.A., Fang X., Salmond G.P.C. (2014). Viunalikeviruses are environmentally common agents of horizontal gene transfer in pathogens and biocontrol bacteria. ISME J..

[104] Matilla M.A., Salmond G.P.C. (2014). Bacteriophage ϕMAM1, a viunalikevirus, is a broad-host-range, high-efficiency generalized transducer that infects environmental and clinical isolates of the enterobacterial genera Serratia and Kluyvera. Appl. Environ. Microbiol..

[105] Kwoh D.Y., Kemper J. (1978). Bacteriophage P22-mediated specialized transduction in Salmonella typhimurium: High frequency of aberrant prophage excision. J. Virol..

[106] Chan R.K., Botstein D. (1976). Specialized transduction by bacteriophage P22 in Salmonella typhimurium: Genetic and physical structure of the transducing genomes and the prophage attachment site. Genetics.

[107] Chan R.K., Botstein D., Watanabe T., Ogata Y. (1972). Specialized transduction of tetracycline resistance by phage P22 in Salmonella typhimurium: II. Properties of a high-frequency-transducing lysate. Virology.

[108] Mise K., Nakaya R. (1977). Transduction of R plasmids by bacteriophages P1 and P22. Mol. Gen. Genet. MGG.

[109] Davidson A.R. (2018). A common trick for transferring bacterial DNA. Science.

[110] Chen J., Quiles-Puchalt N., Chiang Y.N., Bacigalupe R., Fillol-Salom A., Chee M.S.J., Fitzgerald J.R., Penadés J.R. (2018). Genome hypermobility by lateral transduction. Science.

[111] Fillol-Salom A., Alsaadi A., de Sousa J.A.M., Zhong L., Foster K.R., Rocha E.P.C., Penadés J.R., Ingmer H., Haaber J. (2019). Bacteriophages benefit from generalized transduction. PLoS Pathog..

[112] Wendling C.C., Refardt D., Hall A.R. (2020). Fitness benefits to bacteria of carrying prophages and prophage-encoded antibiotic-resistance genes peak in different environments. Evolution.

[113] Taylor V.L., Fitzpatrick A.D., Islam Z., Maxwell K.L., Kielian M., Mettenleiter T.C., Roossinck M.J.B.T.-A. (2019). Chapter One—The Diverse Impacts of Phage Morons on Bacterial Fitness and Virulence.

[114] Abedon S.T., LeJeune J.T. (2005). Why Bacteriophage Encode Exotoxins and other Virulence Factors. Evol. Bioinforma..

[115] Asokan G.V., Ramadhan T., Ahmed E., Sanad H. (2019). WHO global priority pathogens list: A bibliometric analysis of medline-pubmed for knowledge mobilization to infection prevention and control practices in Bahrain. Oman Med. J..

[116] Balcazar J.L. (2014). Bacteriophages as Vehicles for Antibiotic Resistance Genes in the Environment. PLoS Pathog..

[117] Calero-Cáceres W., Ye M., Balcázar J.L. (2019). Bacteriophages as Environmental Reservoirs of Antibiotic Resistance. Trends Microbiol..

[118] Debroas D., Siguret C. (2019). Viruses as key reservoirs of antibiotic resistance genes in the environment. ISME J..

[119] Wang X., Wood T.K. (2016). Cryptic prophages as targets for drug development. Drug Resist. Updat..

[120] Kondo K., Kawano M., Sugai M. (2020). Prophage elements function as reservoir for antibiotic resistance and virulence genes in nosocomial pathogens. bioRxiv.

[121] Enault F., Briet A., Bouteille L., Roux S., Sullivan M.B., Petit M.-A. (2017). Phages rarely encode antibiotic resistance genes: A cautionary tale for virome analyses. ISME J..

[122] Wachino J.-I., Jin W., Kimura K., Arakawa Y. (2019). Intercellular Transfer of Chromosomal Antimicrobial Resistance Genes between Acinetobacter baumannii Strains Mediated by Prophages. Antimicrob. Agents Chemother..

[123] Costa A.R., Monteiro R., Azeredo J. (2018). Genomic analysis of Acinetobacter baumannii prophages reveals remarkable diversity and suggests profound impact on bacterial virulence and fitness. Sci. Rep..

[124] Blahová J., Králiková K., Krčméry V., Ježek P. (2000). Low-Frequency Transduction of Imipenem Resistance and High-Frequency Transduction of Ceftazidime and Aztreonam Resistance by the Bacteriophage AP-151 Isolated from a Pseudomonas aeruginosa Strain. J. Chemother..

[125] Rodríguez-Rubio L., Serna C., Ares-Arroyo M., Matamoros B.R., Delgado-Blas J.F., Montero N., Bernabe-Balas C., Wedel E.F., Mendez I.S., Muniesa M. (2020). Extensive antimicrobial resistance mobilization via multicopy plasmid encapsidation mediated by temperate phages. J. Antimicrob. Chemother..

[126] Lerminiaux N.A., Cameron A.D.S. (2018). Horizontal transfer of antibiotic resistance genes in clinical environments. Can. J. Microbiol..

[127] Mašlaňová I., Stříbná S., Doškař J., Pantůček R. (2016). Efficient plasmid transduction to Staphylococcus aureus strains insensitive to the lytic action of transducing phage. FEMS Microbiol. Lett..

[128] Chlebowicz M.A., Mašlaňová I., Kuntová L., Grundmann H., Pantůček R., Doškař J., van Dijl J.M., Buist G. (2014). The Staphylococcal Cassette Chromosome mec type V from Staphylococcus aureus ST398 is packaged into bacteriophage capsids. Int. J. Med. Microbiol..

[129] Colavecchio A., Cadieux B., Lo A., Goodridge L.D. (2017). Bacteriophages contribute to the spread of antibiotic resistance genes among foodborne pathogens of the Enterobacteriaceae family—A review. Front. Microbiol..

[130] Serra-Moreno R., Acosta S., Hernalsteens J.P., Jofre J., Muniesa M. (2006). Use of the lambda Red recombinase system to produce recombinant prophages carrying antibiotic resistance genes. BMC Mol. Biol..

[131] Shousha A., Awaiwanont N., Sofka D., Smulders F.J.M., Paulsen P., Szostak M.P., Humphrey T., Hilbert F. (2015). Bacteriophages Isolated from Chicken Meat and the Horizontal Transfer of Antimicrobial Resistance Genes. Appl. Environ. Microbiol..

[132] Marinus M.G., Poteete A.R. (2013). High efficiency generalized transduction in Escherichia coli O157:H7. F1000Research.

[133] Schmieger H., Schicklmaier P. (1999). Transduction of multiple drug resistance of Salmonella enterica serovar typhimurium DT104. FEMS Microbiol. Lett..

[134] Bearson B.L., Allen H.K., Brunelle B.W., Lee I.S., Casjens S.R., Stanton T.B. (2014). The agricultural antibiotic carbadox induces phage-mediated gene transfer in Salmonella. Front. Microbiol..

[135] Krahn T., Wibberg D., Maus I., Winkler A., Bontron S., Sczyrba A., Nordmann P., Pühler A., Poirel L., Schlüter A. (2016). Intraspecies Transfer of the Chromosomal Acinetobacter baumannii NDM-1 Carbapenemase Gene. Antimicrob. Agents Chemother..

[136] Brown-Jaque M., Rodriguez Oyarzun L., Cornejo-Sánchez T., Martín-Gómez M.T., Gartner S., de Gracia J., Rovira S., Alvarez A., Jofre J., González-López J.J. (2018). Detection of Bacteriophage Particles Containing Antibiotic Resistance Genes in the Sputum of Cystic Fibrosis Patients. Front. Microbiol..

[137] Stanczak-Mrozek K.I., Manne A., Knight G.M., Gould K., Witney A.A., Lindsay J.A. (2015). Within-host diversity of MRSA antimicrobial resistances. J. Antimicrob. Chemother..

[138] Colomer-Lluch M., Jofre J., Muniesa M. (2014). Quinolone resistance genes (qnrA and qnrS) in bacteriophage particles from wastewater samples and the effect of inducing agents on packaged antibiotic resistance genes. J. Antimicrob. Chemother..

[139] Colomer-Lluch M., Jofre J., Muniesa M. (2011). Antibiotic Resistance Genes in the Bacteriophage DNA Fraction of Environmental Samples. PLoS ONE.

[140] Gómez-Gómez C., Blanco-Picazo P., Brown-Jaque M., Quirós P., Rodríguez-Rubio L., Cerdà-Cuellar M., Muniesa M. (2019). Infectious phage particles packaging antibiotic resistance genes found in meat products and chicken feces. Sci. Rep..

[141] Larrañaga O., Brown-Jaque M., Quirós P., Gómez-Gómez C., Blanch A.R., Rodríguez-Rubio L., Muniesa M. (2018). Phage particles harboring antibiotic resistance genes in fresh-cut vegetables and agricultural soil. Environ. Int..

[142] Gabashvili E., Osepashvili M., Koulouris S., Ujmajuridze L., Tskhitishvili Z., Kotetishvili M. (2020). Phage Transduction is Involved in the Intergeneric Spread of Antibiotic Resistance-Associated blaCTX-M, mel, and tetM Loci in Natural Populations of Some Human and Animal Bacterial Pathogens. Curr. Microbiol..

[143] Bearson B.L., Brunelle B.W. (2015). Fluoroquinolone induction of phage-mediated gene transfer in multidrug-resistant Salmonella. Int. J. Antimicrob. Agents.

[144] Battaglioli E.J., Baisa G.A., Weeks A.E., Schroll R.A., Hryckowian A.J., Welch R.A. (2011). Isolation of generalized transducing bacteriophages for uropathogenic strains of Escherichia coli. Appl. Environ. Microbiol..

[145] Fard R.M.N., Barton M.D., Heuzenroeder M.W. (2011). Bacteriophage-mediated transduction of antibiotic resistance in enterococci. Lett. Appl. Microbiol..

[146] Beutin L., Martin A. (2012). Outbreak of Shiga Toxin-Producing Escherichia coli (STEC) O104:H4 Infection in Germany Causes a Paradigm Shift with Regard to Human Pathogenicity of STEC Strains. J. Food Prot..

[147] Tassinari E., Bawn M., Thilliez G., Charity O., Acton L., Kirkwood M., Petrovska L., Dallman T., Burgess C.M., Hall N. (2020). Whole-genome epidemiology links phage-mediated acquisition of a virulence gene to the clonal expansion of a pandemic Salmonella enterica serovar Typhimurium clone. Microb. Genom..

[148] Bondy-Denomy J., Qian J., Westra E.R., Buckling A., Guttman D.S., Davidson A.R., Maxwell K.L. (2016). Prophages mediate defense against phage infection through diverse mechanisms. ISME J..

[149] Davies E.V., Winstanley C., Fothergill J.L., James C.E. (2016). The role of temperate bacteriophages in bacterial infection. FEMS Microbiol. Lett..

[150] Sabour P.M., Griffiths M. (2010). Bacteriophages in the Control of Food- and Waterborne Pathogens.

[151] Hofer B., Ruge M., Dreiseikelmann B. (1995). The superinfection exclusion gene (sieA) of bacteriophage P22: Identification and overexpression of the gene and localization of the gene product. J. Bacteriol..

[152] Nesper J., Blass J., Fountoulakis M., Reidl J. (1999). Characterization of the major control region of Vibrio cholerae bacteriophage K139: Immunity, exclusion, and integration. J. Bacteriol..

[153] Groth A.C., Calos M.P. (2004). Phage Integrases: Biology and Applications. J. Mol. Biol..

[154] Fogg P.C.M., Colloms S., Rosser S., Stark M., Smith M.C.M. (2014). New applications for phage integrases. J. Mol. Biol..

[155] Marchler-Bauer A., Bo Y., Han L., He J., Lanczycki C.J., Lu S., Chitsaz F., Derbyshire M.K., Geer R.C., Gonzales N.R. (2017). CDD/SPARCLE: Functional classification of proteins via subfamily domain architectures. Nucleic Acids Res..

[156] Carnoy C., Roten C.-A. (2009). The dif/Xer Recombination Systems in Proteobacteria. PLoS ONE.

[157] Van Houdt R., Leplae R., Lima-Mendez G., Mergeay M., Toussaint A. (2012). Towards a more accurate annotation of tyrosine-based site-specific recombinases in bacterial genomes. Mob. DNA.

[158] Lunt B.L., Hatfull G.F. (2016). Brujita Integrase: A Simple, Arm-Less, Directionless, and Promiscuous Tyrosine Integrase System. J. Mol. Biol..

[159] Smyshlyaev G., Barabas O., Bateman A. (2019). Sequence analysis allows functional annotation of tyrosine recombinases in prokaryotic genomes. bioRxiv.

[160] Wozniak R.A.F., Waldor M.K. (2010). Integrative and conjugative elements: Mosaic mobile genetic elements enabling dynamic lateral gene flow. Nat. Rev. Microbiol..

[161] Campbell A. (2003). Prophage insertion sites. Res. Microbiol..

[162] Burrus V., Waldor M.K. (2003). Control of SXT Integration and Excision. J. Bacteriol..

[163] Boyd E.F., Almagro-Moreno S., Parent M.A. (2009). Genomic islands are dynamic, ancient integrative elements in bacterial evolution. Trends Microbiol..

[164] Johnson C.M., Grossman A.D. (2015). Integrative and Conjugative Elements (ICEs): What They Do and How They Work. Annu. Rev. Genet..

[165] Suzuki S., Yoshikawa M., Imamura D., Abe K., Eichenberger P., Sato T. (2020). Compatibility of Site-Specific Recombination Units between Mobile Genetic Elements. iScience.

[166] Cury J., Touchon M., Rocha E.P.C. (2017). Integrative and conjugative elements and their hosts: Composition, distribution and organization. Nucleic Acids Res..

[167] Palmieri C., Varaldo P.E., Facinelli B. (2011). Streptococcus suis, an Emerging Drug-Resistant Animal and Human Pathogen. Front. Microbiol..

[168] Gilcrease E.B., Casjens S.R. (2018). The genome sequence of Escherichia coli tailed phage D6 and the diversity of Enterobacteriales circular plasmid prophages. Virology.

[169] Cury J., Oliveira P.H., de la Cruz F., Rocha E.P.C. (2018). Host Range and Genetic Plasticity Explain the Coexistence of Integrative and Extrachromosomal Mobile Genetic Elements. Mol. Biol. Evol..

[170] Partridge S.R., Kwong S.M., Firth N., Jensen S.O. (2018). Mobile Genetic Elements Associated with Antimicrobial Resistance. Clin. Microbiol. Rev..

[171] Silva C., Calva E., Fernández-Mora M., Puente J.L., Vinuesa P. (2019). Population analysis of D6-like plasmid prophage variants associated with specific IncC plasmid types in the emerging Salmonella Typhimurium ST213 genotype. PLoS ONE.

[172] Fillol-Salom A., Martínez-Rubio R., Abdulrahman R.F., Chen J., Davies R., Penadés J.R. (2018). Phage-inducible chromosomal islands are ubiquitous within the bacterial universe. ISME J..

[173] Ibarra-Chávez R., Haag A.F., Dorado-Morales P., Lasa I., Penadés J.R. (2020). Rebooting Synthetic Phage-Inducible Chromosomal Islands: One Method to Forge Them All. BioDesign Res..

[174] Fortier L.-C., Sekulovic O. (2013). Importance of prophages to evolution and virulence of bacterial pathogens. Virulence.

[175] Li G., Cortez M.H., Dushoff J., Weitz J.S. (2020). When to be Temperate: On the Fitness Benefits of Lysis vs. Lysogeny. bioRxiv.

[176] Bossi L., Fuentes J.A., Mora G., Figueroa-Bossi N. (2003). Prophage contribution to bacterial population dynamics. J. Bacteriol..

[177] Hyman P. (2019). Phages for Phage Therapy: Isolation, Characterization, and Host Range Breadth. Pharmaceuticals.

[178] Monteiro R., Pires D.P., Costa A.R., Azeredo J. (2019). Phage Therapy: Going Temperate?. Trends Microbiol..

[179] Górski A., Borysowski J., Międzybrodzki R. (2020). Phage Therapy: Towards a Successful Clinical Trial. Antibiotics.

[180] Stern A., Sorek R. (2011). The phage-host arms race: Shaping the evolution of microbes. Bioessays.

[181] Labrie S.J., Samson J.E., Moineau S. (2010). Bacteriophage resistance mechanisms. Nat. Rev. Microbiol..

[182] Chan B.K., Abedon S.T., Loc-Carrillo C. (2013). Phage cocktails and the future of phage therapy. Future Microbiol..

[183] Oechslin F. (2018). Resistance Development to Bacteriophages Occurring during Bacteriophage Therapy. Viruses.

[184] Jończyk E., Kłak M., Międzybrodzki R., Górski A. (2011). The influence of external factors on bacteriophages—review. Folia Microbiol..

[185] Malik D.J., Sokolov I.J., Vinner G.K., Mancuso F., Cinquerrui S., Vladisavljevic G.T., Clokie M.R.J., Garton N.J., Stapley A.G.F., Kirpichnikova A. (2017). Formulation, stabilisation and encapsulation of bacteriophage for phage therapy. Adv. Colloid Interface Sci..

[186] Petsong K., Benjakul S., Vongkamjan K. (2020). Optimization of wall material for phage encapsulation via freeze-drying and antimicrobial efficacy of microencapsulated phage against Salmonella. J. Food Sci. Technol..

[187] Loh B., Gondil V.S., Manohar P., Khan F.M., Yang H., Leptihn S. (2020). Encapsulation and Delivery of Therapeutic Phages. Appl. Environ. Microbiol..

[188] Lone A., Anany H., Hakeem M., Aguis L., Avdjian A.-C., Bouget M., Atashi A., Brovko L., Rochefort D., Griffiths M.W. (2016). Development of prototypes of bioactive packaging materials based on immobilized bacteriophages for control of growth of bacterial pathogens in foods. Int. J. Food Microbiol..

[189] Kering K.K., Zhang X., Nyaruaba R., Yu J., Wei H. (2020). Application of adaptive evolution to improve the stability of bacteriophages during storage. Viruses.

[190] Favor A.H., Llanos C.D., Youngblut M.D., Bardales J.A. (2020). Optimizing bacteriophage engineering through an accelerated evolution platform. Sci. Rep..

[191] Carlton R.M., Noordman W.H., Biswas B., de Meester E.D., Loessner M.J. (2005). Bacteriophage P100 for control of Listeria monocytogenes in foods: Genome sequence, bioinformatic analyses, oral toxicity study, and application. Regul. Toxicol. Pharmacol..

[192] Abedon S.T. (2014). Phage Therapy: Eco-Physiological Pharmacology. Scientifica.

[193] Luong T., Salabarria A.-C., Edwards R.A., Roach D.R. (2020). Standardized bacteriophage purification for personalized phage therapy. Nat. Protoc..

[194] Melo L.D.R., Oliveira H., Pires D.P., Dabrowska K., Azeredo J. (2020). Phage therapy efficacy: A review of the last 10 years of preclinical studies. Crit. Rev. Microbiol..

[195] Luong T., Salabarria A.-C., Roach D.R. (2020). Phage Therapy in the Resistance Era: Where Do We Stand and Where Are We Going?. Clin. Ther..

[196] Ding C., He J. (2010). Effect of antibiotics in the environment on microbial populations. Appl. Microbiol. Biotechnol..

[197] Muniesa M., Imamovic L., Jofre J. (2011). Bacteriophages and genetic mobilization in sewage and faecally polluted environments. Microb. Biotechnol..

[198] Brown T.L., Petrovski S., Chan H.T., Angove M.J., Tucci J. (2018). Semi-Solid and Solid Dosage Forms for the Delivery of Phage Therapy to Epithelia. Pharmaceuticals.

[199] Brown T.L., Petrovski S., Hoyle D., Chan H.T., Lock P., Tucci J. (2017). Characterization and formulation into solid dosage forms of a novel bacteriophage lytic against Klebsiella oxytoca. PLoS ONE.

[200] Vikram A., Woolston J., Sulakvelidze A. (2020). Phage Biocontrol Applications in Food Production and Processing. Bact. Viruses Exploit. Biocontrol Ther..

[201] Moye Z.D., Woolston J., Sulakvelidze A. (2018). Bacteriophage Applications for Food Production and Processing. Viruses.

[202] Bintsis T. (2017). Foodborne pathogens. AIMS Microbiol..

[203] Government of Canada Surveillance of Salmonellosis (Salmonella). https://www.canada.ca/en/public-health/services/diseases/salmonellosis-salmonella/surveillance.html.

[204] Centers for Disease Control and Prevention Highlights from the 2017 Surveillance Report|Foodborne Disease Outbreak Surveillance System. https://www.cdc.gov/fdoss/annual-reports/2017-report-highlights.html.

[205] Centers for Disease Control and Prevention (2012). National Shiga Toxin-Producing Escherichia coli (STEC) Surveillance Overview.

[206] Guenther S., Huwyler D., Richard S., Loessner M.J. (2009). Virulent Bacteriophage for Efficient Biocontrol of Listeria monocytogenes in Ready-To-Eat Foods. Appl. Environ. Microbiol..

[207] Perera M.N., Abuladze T., Li M., Woolston J., Sulakvelidze A. (2015). Bacteriophage cocktail significantly reduces or eliminates Listeria monocytogenes contamination on lettuce, apples, cheese, smoked salmon and frozen foods. Food Microbiol..

[208] Figueiredo A.C.L., Almeida R.C.C. (2017). Antibacterial efficacy of nisin, bacteriophage P100 and sodium lactate against Listeria monocytogenes in ready-to-eat sliced pork ham. Braz. J. Microbiol..

[209] Holck A., Berg J. (2009). Inhibition of Listeria monocytogenes in cooked ham by virulent bacteriophages and protective cultures. Appl. Environ. Microbiol..

[210] Zhang X., Niu Y.D., Nan Y., Stanford K., Holley R., McAllister T., Narváez-Bravo C. (2019). SalmoFresh^TM^ effectiveness in controlling Salmonella on romaine lettuce, mung bean sprouts and seeds. Int. J. Food Microbiol..

[211] Sharma C.S., Dhakal J., Nannapaneni R. (2015). Efficacy of Lytic Bacteriophage Preparation in Reducing Salmonella In Vitro, on Turkey Breast Cutlets, and on Ground Turkey. J. Food Prot..

[212] Sukumaran A.T., Nannapaneni R., Kiess A., Sharma C.S. (2016). Reduction of Salmonella on chicken breast fillets stored under aerobic or modified atmosphere packaging by the application of lytic bacteriophage preparation SalmoFreshTM1,2. Poult. Sci..

[213] Zampara A., Sørensen M.C.H., Elsser-Gravesen A., Brøndsted L. (2017). Significance of phage-host interactions for biocontrol of Campylobacter jejuni in food. Food Control.

[214] Bigwood T., Hudson J.A., Billington C., Carey-Smith G.V., Heinemann J.A. (2008). Phage inactivation of foodborne pathogens on cooked and raw meat. Food Microbiol..

[215] O’Flynn G., Ross R.P., Fitzgerald G.F., Coffey A. (2004). Evaluation of a cocktail of three bacteriophages for biocontrol of Escherichia coli O157:H7. Appl. Environ. Microbiol..

[216] Abuladze T., Li M., Menetrez M.Y., Dean T., Senecal A., Sulakvelidze A. (2008). Bacteriophages Reduce Experimental Contamination of Hard Surfaces, Tomato, Spinach, Broccoli, and Ground Beef by Escherichia coli O157:H7. Appl. Environ. Microbiol..

[217] Greer G.G. (1982). Psychrotrophic Bacteriophages for Beef Spoilage Pseudomonads1. J. Food Prot..

[218] Snyder A.B., Perry J.J., Yousef A.E. (2016). Developing and optimizing bacteriophage treatment to control enterohemorrhagic Escherichia coli on fresh produce. Int. J. Food Microbiol..

[219] Leverentz B., Conway W.S., Camp M.J., Janisiewicz W.J., Abuladze T., Yang M., Saftner R., Sulakvelidze A. (2003). Biocontrol of Listeria monocytogenes on Fresh-Cut Produce by Treatment with Lytic Bacteriophages and a Bacteriocin. Appl. Environ. Microbiol..

[220] Leverentz B., Conway W.S., Janisiewicz W., Camp M.J. (2004). Optimizing Concentration and Timing of a Phage Spray Application To Reduce Listeria monocytogenes on Honeydew Melon Tissue. J. Food Prot..

[221] Buttimer C., McAuliffe O., Ross R.P., Hill C., O’Mahony J., Coffey A. (2017). Bacteriophages and Bacterial Plant Diseases. Front. Microbiol..

[222] Gill J.J., Svircev A.M., Smith R., Castle A.J. (2003). Bacteriophages of Erwinia amylovora. Appl. Environ. Microbiol..

[223] Gayder S., Parcey M., Nesbitt D., Castle A.J., Svircev A.M. (2020). Population Dynamics between Erwinia amylovora, Pantoea agglomerans and Bacteriophages: Exploiting Synergy and Competition to Improve Phage Cocktail Efficacy. Microorganisms.

[224] Svircev A.M., Anany H., Wang Q., Castle A.J. Successful Control of Fire Blight: Can Bacteriophages do the Job?. Proceedings of the Fourth International Symposium on Biological Control of Bacterial Plant Diseases.

[225] Roach D.R., Lehman S.M., Castle A.J., Svircev A.M. A Bacteriophage-Based Biopesticide to Control Fire Blight. Proceedings of the Annual Meeting and Exhibition.

[226] Nagai H., Miyake N., Kato S., Maekawa D., Inoue Y., Takikawa Y. (2017). Improved control of black rot of broccoli caused by Xanthomonas campestris pv. campestris using a bacteriophage and a nonpathogenic Xanthomonas sp. strain. J. Gen. Plant Pathol..

[227] Ramírez M., Neuman B.W., Ramírez C.A. (2020). Bacteriophages as promising agents for the biological control of Moko disease (Ralstonia solanacearum) of banana. Biol. Control.

[228] Jones J.B., Vallad G.E., Iriarte F.B., Obradović A., Wernsing M.H., Jackson L.E., Balogh B., Hong J.C., Momol M.T. (2012). Considerations for using bacteriophages for plant disease control. Bacteriophage.

[229] Vu N.T., Oh C.-S. (2020). Bacteriophage Usage for Bacterial Disease Management and Diagnosis in Plants. Plant Pathol. J..

[230] Svircev A., Roach D., Castle A. (2018). Framing the Future with Bacteriophages in Agriculture. Viruses.

[231] Holtappels D., Fortuna K., Lavigne R., Wagemans J. (2021). The future of phage biocontrol in integrated plant protection for sustainable crop production. Curr. Opin. Biotechnol..

[232] Sieiro C., Areal-Hermida L., Pichardo-Gallardo Á., Almuiña-González R., de Miguel T., Sánchez S., Sánchez-Pérez Á., Villa T.G. (2020). A Hundred Years of Bacteriophages: Can Phages Replace Antibiotics in Agriculture and Aquaculture?. Antibiotics.

[233] Culot A., Grosset N., Gautier M. (2019). Overcoming the challenges of phage therapy for industrial aquaculture: A review. Aquaculture.

[234] Żaczek M., Weber-Dąbrowska B., Górski A. (2020). Phages as a Cohesive Prophylactic and Therapeutic Approach in Aquaculture Systems. Antibiotics.

[235] Folsom J.P., Frank J.F. (2006). Chlorine Resistance of Listeria monocytogenes Biofilms and Relationship to Subtype, Cell Density, and Planktonic Cell Chlorine Resistance. J. Food Prot..

[236] Melo L.D.R., Ferreira R., Costa A.R., Oliveira H., Azeredo J. (2019). Efficacy and safety assessment of two enterococci phages in an in vitro biofilm wound model. Sci. Rep..

[237] Łusiak-Szelachowska M., Weber-Dąbrowska B., Górski A. (2020). Bacteriophages and Lysins in Biofilm Control. Virol. Sin..

[238] Abedon S.T., García P., Mullany P., Aminov R. (2017). Editorial: Phage therapy: Past, present and future. Front. Microbiol..

[239] Oechslin F., Piccardi P., Mancini S., Gabard J., Moreillon P., Entenza J.M., Resch G., Que Y.-A. (2017). Synergistic Interaction Between Phage Therapy and Antibiotics Clears Pseudomonas Aeruginosa Infection in Endocarditis and Reduces Virulence. J. Infect. Dis..

[240] Melo L.D.R., Veiga P., Cerca N., Kropinski A.M., Almeida C., Azeredo J., Sillankorva S. (2016). Development of a Phage Cocktail to Control Proteus mirabilis Catheter-associated Urinary Tract Infections. Front. Microbiol..

[241] Nzakizwanayo J., Hanin A., Alves D.R., McCutcheon B., Dedi C., Salvage J., Knox K., Stewart B., Metcalfe A., Clark J. (2016). Bacteriophage Can Prevent Encrustation and Blockage of Urinary Catheters by Proteus mirabilis. Antimicrob. Agents Chemother..

[242] Morris J., Kelly N., Elliott L., Grant A., Wilkinson M., Hazratwala K., McEwen P. (2018). Evaluation of Bacteriophage Anti-Biofilm Activity for Potential Control of Orthopedic Implant-Related Infections Caused by Staphylococcus aureus. Surg. Infect..

[243] Khalifa L., Brosh Y., Gelman D., Coppenhagen-Glazer S., Beyth S., Poradosu-Cohen R., Que Y.-A., Beyth N., Hazan R. (2015). Targeting Enterococcus faecalis biofilms with phage therapy. Appl. Environ. Microbiol..

[244] Shen Y., Loessner M.J. (2021). Beyond antibacterials—Exploring bacteriophages as antivirulence agents. Curr. Opin. Biotechnol..

[245] Knecht L.E., Veljkovic M., Fieseler L. (2020). Diversity and Function of Phage Encoded Depolymerases. Front. Microbiol..

[246] Ferriol-González C., Domingo-Calap P. (2020). Phages for Biofilm Removal. Antibiotics.

[247] Gray J.A., Chandry P.S., Kaur M., Kocharunchitt C., Bowman J.P., Fox E.M. (2018). Novel Biocontrol Methods for Listeria monocytogenes Biofilms in Food Production Facilities. Front. Microbiol..

[248] Gutiérrez D., Rodríguez-Rubio L., Martínez B., Rodríguez A., García P. (2016). Bacteriophages as Weapons Against Bacterial Biofilms in the Food Industry. Front. Microbiol..

[249] Furfaro L.L., Payne M.S., Chang B.J. (2018). Bacteriophage Therapy: Clinical Trials and Regulatory Hurdles. Front. Cell. Infect. Microbiol..

[250] Rhoads D.D., Wolcott R.D., Kuskowski M.A., Wolcott B.M., Ward L.S., Sulakvelidze A. (2009). Bacteriophage therapy of venous leg ulcers in humans: Results of a phase I safety trial. J. Wound Care.

[251] Wright A., Hawkins C.H., Änggård E.E., Harper D.R. (2009). A controlled clinical trial of a therapeutic bacteriophage preparation in chronic otitis due to antibiotic-resistant Pseudomonas aeruginosa; a preliminary report of efficacy. Clin. Otolaryngol..

[252] Fabijan A.P., Lin R.C.Y., Ho J., Maddocks S., Zakour N.L.B., Iredell J.R., Khalid A., Venturini C., Chard R., Morales S. (2020). Safety of bacteriophage therapy in severe Staphylococcus aureus infection. Nat. Microbiol..

[253] Jikia D., Chkhaidze N., Imedashvili E., Mgaloblishvili I., Tsitlanadze G., Katsarava R., Morris J.G., Sulakvelidze A. (2005). The use of a novel biodegradable preparation capable of the sustained release of bacteriophages and ciprofloxacin, in the complex treatment of multidrug-resistant Staphylococcus aureus-infected local radiation injuries caused by exposure to Sr90. Clin. Exp. Dermatol..

[254] Schooley R.T., Biswas B., Gill J.J., Hernandez-Morales A., Lancaster J., Lessor L., Barr J.J., Reed S.L., Rohwer F., Benler S. (2017). Development and Use of Personalized Bacteriophage-Based Therapeutic Cocktails To Treat a Patient with a Disseminated Resistant Acinetobacter baumannii. Antimicrob. Agents Chemother..

[255] Cha K., Oh H.K., Jang J.Y., Jo Y., Kim W.K., Ha G.U., Ko K.S., Myung H. (2018). Characterization of Two Novel Bacteriophages Infecting Multidrug-Resistant (MDR) Acinetobacter baumannii and Evaluation of Their Therapeutic Efficacy in Vivo. Front. Microbiol..

[256] Roach D.R., Leung C.Y., Henry M., Morello E., Singh D., Di Santo J.P., Weitz J.S., Debarbieux L. (2017). Synergy between the Host Immune System and Bacteriophage Is Essential for Successful Phage Therapy against an Acute Respiratory Pathogen. Cell Host Microbe.

[257] Fish R., Kutter E., Wheat G., Blasdel B., Kutateladze M., Kuhl S. (2016). Bacteriophage treatment of intransigent diabetic toe ulcers: A case series. J. Wound Care.

[258] McCallin S., Sacher J.C., Zheng J., Chan B.K. (2019). Current State of Compassionate Phage Therapy. Viruses.

[259] Allen R.C., Pfrunder-Cardozo K.R., Meinel D., Egli A., Hall A.R. (2017). Associations among Antibiotic and Phage Resistance Phenotypes in Natural and Clinical Escherichia coli Isolates. MBio.

[260] Burmeister A.R., Fortier A., Roush C., Lessing A.J., Bender R.G., Barahman R., Grant R., Chan B.K., Turner P.E. (2020). Pleiotropy complicates a trade-off between phage resistance and antibiotic resistance. Proc. Natl. Acad. Sci. USA.

[261] Comeau A.M., Tétart F., Trojet S.N., Prère M.-F., Krisch H.M. (2007). Phage-Antibiotic Synergy (PAS): β-Lactam and Quinolone Antibiotics Stimulate Virulent Phage Growth. PLoS ONE.

[262] Tagliaferri T.L., Jansen M., Horz H.-P. (2019). Fighting Pathogenic Bacteria on Two Fronts: Phages and Antibiotics as Combined Strategy. Front. Cell. Infect. Microbiol..

[263] Valério N., Oliveira C., Jesus V., Branco T., Pereira C., Moreirinha C., Almeida A. (2017). Effects of single and combined use of bacteriophages and antibiotics to inactivate Escherichia coli. Virus Res..

[264] Rahman M., Kim S., Kim S.M., Seol S.Y., Kim J. (2011). Characterization of induced Staphylococcus aureus bacteriophage SAP-26 and its anti-biofilm activity with rifampicin. Biofouling.

[265] Torres-Barceló C., Arias-Sánchez F.I., Vasse M., Ramsayer J., Kaltz O., Hochberg M.E. (2014). A Window of Opportunity to Control the Bacterial Pathogen Pseudomonas aeruginosa Combining Antibiotics and Phages. PLoS ONE.

[266] Canfield G.S., Chatterjee A., Mangalea M.R., Sheriff E.K., Keidan M., McBride S.W., McCollister B.D., Duerkop B.A. (2020). Lytic bacteriophages facilitate antibiotic sensitization of Enterococcus faecium. bioRxiv.

[267] Jeon J., Park J.-H., Yong D. (2019). Efficacy of bacteriophage treatment against carbapenem-resistant Acinetobacter baumannii in Galleria mellonella larvae and a mouse model of acute pneumonia. BMC Microbiol..

[268] LaVergne S., Hamilton T., Biswas B., Kumaraswamy M., Schooley R.T., Wooten D. (2018). Phage Therapy for a Multidrug-Resistant Acinetobacter baumannii Craniectomy Site Infection. Open Forum Infect. Dis..

[269] Cafora M., Deflorian G., Forti F., Ferrari L., Binelli G., Briani F., Ghisotti D., Pistocchi A. (2019). Phage therapy against Pseudomonas aeruginosa infections in a cystic fibrosis zebrafish model. Sci. Rep..

[270] de Melo A.C.C., da Mata Gomes A., Melo F.L., Ardisson-Araújo D.M.P., de Vargas A.P.C., Ely V.L., Kitajima E.W., Ribeiro B.M., Wolff J.L.C. (2019). Characterization of a bacteriophage with broad host range against strains of Pseudomonas aeruginosa isolated from domestic animals. BMC Microbiol..

[271] Kwiatek M., Parasion S., Rutyna P., Mizak L., Gryko R., Niemcewicz M., Olender A., Łobocka M. (2017). Isolation of bacteriophages and their application to control Pseudomonas aeruginosa in planktonic and biofilm models. Res. Microbiol..

[272] Titze I., Lehnherr T., Lehnherr H., Krömker V. (2020). Efficacy of Bacteriophages Against Staphylococcus aureus Isolates from Bovine Mastitis. Pharmaceuticals.

[273] Dissanayake U., Ukhanova M., Moye Z.D., Sulakvelidze A., Mai V. (2019). Bacteriophages Reduce Pathogenic Escherichia coli Counts in Mice Without Distorting Gut Microbiota. Front. Microbiol..

[274] Cieplak T., Soffer N., Sulakvelidze A., Nielsen D.S. (2018). A bacteriophage cocktail targeting Escherichia coli reduces E. coli in simulated gut conditions, while preserving a non-targeted representative commensal normal microbiota. Gut Microbes.

[275] Lukman C., Yonathan C., Magdalena S., Waturangi D.E. (2020). Isolation and characterization of pathogenic Escherichia coli bacteriophages from chicken and beef offal. BMC Res. Notes.

[276] Islam M.S., Zhou Y., Liang L., Nime I., Liu K., Yan T., Wang X., Li J. (2019). Application of a Phage Cocktail for Control of Salmonella in Foods and Reducing Biofilms. Viruses.

[277] Hungaro H.M., Mendonça R.C.S., Gouvêa D.M., Vanetti M.C.D., de Pinto C.L.O. (2013). Use of bacteriophages to reduce Salmonella in chicken skin in comparison with chemical agents. Food Res. Int..

[278] Kittler S., Fischer S., Abdulmawjood A., Glünder G., Klein G. (2013). Effect of bacteriophage application on Campylobacter jejuni loads in commercial broiler flocks. Appl. Environ. Microbiol..

[279] Anany H., Chou Y., Cucic S., Derda R., Evoy S., Griffiths M.W. (2017). From Bits and Pieces to Whole Phage to Nanomachines: Pathogen Detection Using Bacteriophages. Annu. Rev. Food Sci. Technol..

[280] Wisuthiphaet N., Yang X., Young G.M., Nitin N. (2019). Rapid detection of Escherichia coli in beverages using genetically engineered bacteriophage T7. AMB Express.

[281] Richter Ł., Janczuk-Richter M., Niedziółka-Jönsson J., Paczesny J., Hołyst R. (2018). Recent advances in bacteriophage-based methods for bacteria detection. Drug Discov. Today.

[282] Schmelcher M., Loessner M.J. (2014). Application of bacteriophages for detection of foodborne pathogens. Bacteriophage.

[283] Schofield D., Sharp N.J., Westwater C. (2012). Phage-based platforms for the clinical detection of human bacterial pathogens. Bacteriophage.

[284] Schenborn E., Groskreutz D. (1999). Reporter gene vectors and assays. Mol. Biotechnol..

[285] Meile S., Kilcher S., Loessner M.J., Dunne M. (2020). Reporter Phage-Based Detection of Bacterial Pathogens: Design Guidelines and Recent Developments. Viruses.

[286] Šuster K., Podgornik A., Cör A. (2017). Quick bacteriophage-mediated bioluminescence assay for detecting Staphylococcus spp. in sonicate fluid of orthopaedic artificial joints. New Microbiol..

[287] Meile S., Sarbach A., Du J., Schuppler M., Saez C., Loessner M.J., Kilcher S. (2020). Engineered Reporter Phages for Rapid Bioluminescence-Based Detection and Differentiation of Viable Listeria Cells. Appl. Environ. Microbiol..

[288] Kim S., Kim M., Ryu S. (2014). Development of an Engineered Bioluminescent Reporter Phage for the Sensitive Detection of Viable Salmonella Typhimurium. Anal. Chem..

[289] Ripp S., Jegier P., Johnson C.M., Brigati J.R., Sayler G.S. (2008). Bacteriophage-amplified bioluminescent sensing of Escherichia coli O157:H7. Anal. Bioanal. Chem..

[290] Vinay M., Franche N., Grégori G., Fantino J.-R., Pouillot F., Ansaldi M. (2015). Phage-Based Fluorescent Biosensor Prototypes to Specifically Detect Enteric Bacteria Such as E. coli and Salmonella enterica Typhimurium. PLoS ONE.

[291] Chang T.C., Ding H.C., Chen S. (2002). A Conductance Method for the Identification of Escherichia coli O157:H7 Using Bacteriophage AR1. J. Food Prot..

[292] Swift B.M.C., Meade N., Barron E.S., Bennett M., Perehenic T., Hughes V., Stevenson K., Rees C.E.D. (2020). The development and use of Actiphage^®^ to detect viable mycobacteria from bovine tuberculosis and Johne’s disease-infected animals. Microb. Biotechnol..

[293] Tawil N., Sacher E., Mandeville R., Meunier M. (2012). Surface plasmon resonance detection of E. coli and methicillin-resistant S. aureus using bacteriophages. Biosens. Bioelectron..

[294] Schmidt A., Rabsch W., Broeker N.K., Barbirz S. (2016). Bacteriophage tailspike protein based assay to monitor phase variable glucosylations in Salmonella O-antigens. BMC Microbiol..

[295] Sumrall E.T., Röhrig C., Hupfeld M., Selvakumar L., Du J., Dunne M., Schmelcher M., Shen Y., Loessner M.J. (2020). Glycotyping and Specific Separation of Listeria monocytogenes with a Novel Bacteriophage Protein Tool Kit. Appl. Environ. Microbiol..

[296] Singh A., Arya S.K., Glass N., Hanifi-Moghaddam P., Naidoo R., Szymanski C.M., Tanha J., Evoy S. (2010). Bacteriophage tailspike proteins as molecular probes for sensitive and selective bacterial detection. Biosens. Bioelectron..

[297] Javed M.A., Poshtiban S., Arutyunov D., Evoy S., Szymanski C.M. (2013). Bacteriophage Receptor Binding Protein Based Assays for the Simultaneous Detection of Campylobacter jejuni and Campylobacter coli. PLoS ONE.

[298] Gu J., Lu R., Liu X., Han W., Lei L., Gao Y., Zhao H., Li Y., Diao Y. (2011). LysGH15B, the SH3b Domain of Staphylococcal Phage Endolysin LysGH15, Retains High Affinity to Staphylococci. Curr. Microbiol..

[299] Loessner M.J., Kramer K., Ebel F., Scherer S. (2002). C-terminal domains of Listeria monocytogenes bacteriophage murein hydrolases determine specific recognition and high-affinity binding to bacterial cell wall carbohydrates. Mol. Microbiol..

[300] Kretzer J.W., Schmelcher M., Loessner M.J. (2018). Ultrasensitive and Fast Diagnostics of Viable Listeria Cells by CBD Magnetic Separation Combined with A511::luxAB Detection. Viruses.

[301] Kutter E.M., Kuhl S.J., Abedon S.T. (2015). Re-establishing a place for phage therapy in western medicine. Future Microbiol..

[302] Hagens S., Loessner M.J. (2010). Bacteriophage for biocontrol of foodborne pathogens: Calculations and considerations. Curr. Pharm. Biotechnol..

[303] Chibani-Chennoufi S., Bruttin A., Dillmann M.-L., Brüssow H. (2004). Phage-Host Interaction: An Ecological Perspective. J. Bacteriol..

[304] De Smet J., Hendrix H., Blasdel B.G., Danis-Wlodarczyk K., Lavigne R. (2017). Pseudomonas predators: Understanding and exploiting phage-host interactions. Nat. Rev. Microbiol..

[305] Stone E., Campbell K., Grant I., McAuliffe O. (2019). Understanding and Exploiting Phage-Host Interactions. Viruses.

[306] Gilbert R.A., Kelly W.J., Altermann E., Leahy S.C., Minchin C., Ouwerkerk D., Klieve A.V. (2017). Toward Understanding Phage:Host Interactions in the Rumen; Complete Genome Sequences of Lytic Phages Infecting Rumen Bacteria. Front. Microbiol..

[307] Gill J., Hyman P. (2010). Phage choice, isolation, and preparation for phage therapy. Curr. Pharm. Biotechnol..

[308] Russell D.A., Clokie M.R.J., Kropinski A.M., Lavigne R. (2018). Sequencing, Assembling, and Finishing Complete Bacteriophage Genomes BT—Bacteriophages: Methods and Protocols.

[309] Shkoporov A.N., Ryan F.J., Draper L.A., Forde A., Stockdale S.R., Daly K.M., McDonnell S.A., Nolan J.A., Sutton T.D.S., Dalmasso M. (2018). Reproducible protocols for metagenomic analysis of human faecal phageomes. Microbiome.

[310] Shkoporov A.N., Hill C. (2019). Bacteriophages of the human gut: The “known unknown” of the microbiome. Cell Host Microbe.

[311] De Sordi L., Lourenço M., Debarbieux L. (2019). The battle within: Interactions of bacteriophages and bacteria in the gastrointestinal tract. Cell Host Microbe.

[312] Norman J.M., Handley S.A., Baldridge M.T., Droit L., Liu C.Y., Keller B.C., Kambal A., Monaco C.L., Zhao G., Fleshner P. (2015). Disease-specific alterations in the enteric virome in inflammatory bowel disease. Cell.

[313] Monaco C.L., Gootenberg D.B., Zhao G., Handley S.A., Ghebremichael M.S., Lim E.S., Lankowski A., Baldridge M.T., Wilen C.B., Flagg M. (2016). Altered virome and bacterial microbiome in human immunodeficiency virus-associated acquired immunodeficiency syndrome. Cell Host Microbe.

[314] Reyes A., Blanton L.V., Cao S., Zhao G., Manary M., Trehan I., Smith M.I., Wang D., Virgin H.W., Rohwer F. (2015). Gut DNA viromes of Malawian twins discordant for severe acute malnutrition. Proc. Natl. Acad. Sci. USA.

[315] Bikel S., López-Leal G., Cornejo-Granados F., Gallardo-Becerra L., Sánchez F., Equihua-Medina E., Ochoa-Romo J.P., López-Contreras B.E., Canizales-Quinteros S., Leyva A.O. (2020). Gut Phageome Analysis Reveals Disease-Specific Hallmarks in Childhood Obesity. bioRxiv.

[316] Kim B.O., Kim E.S., Yoo Y.J., Bae H.W., Chung I.Y., Cho Y.H. (2019). Phage-derived antibacterials: Harnessing the simplicity, plasticity, and diversity of phages. Viruses.

[317] Lin T.-Y., Lo Y.-H., Tseng P.-W., Chang S.-F., Lin Y.-T., Chen T.-S. (2012). A T3 and T7 Recombinant Phage Acquires Efficient Adsorption and a Broader Host Range. PLoS ONE.

[318] Dunne M., Rupf B., Tala M., Qabrati X., Ernst P., Shen Y., Sumrall E., Heeb L., Plückthun A., Loessner M.J. (2019). Reprogramming Bacteriophage Host Range through Structure-Guided Design of Chimeric Receptor Binding Proteins. Cell Rep..

[319] Schmelcher M., Donovan D.M., Loessner M.J. (2012). Bacteriophage endolysins as novel antimicrobials. Future Microbiol..

[320] Gerstmans H., Rodríguez-Rubio L., Lavigne R., Briers Y. (2016). From endolysins to Artilysin^®^s: Novel enzyme-based approaches to kill drug-resistant bacteria. Biochem. Soc. Trans..

[321] Zhou B., Zhen X., Zhou H., Zhao F., Fan C., Perčulija V., Tong Y., Mi Z., Ouyang S. (2020). Structural and functional insights into a novel two-component endolysin encoded by a single gene in Enterococcus faecalis phage. PLOS Pathog..

[322] Plotka M., Sancho-Vaello E., Dorawa S., Kaczorowska A.-K., Kozlowski L.P., Kaczorowski T., Zeth K. (2019). Structure and function of the Ts2631 endolysin of Thermus scotoductus phage vB_Tsc2631 with unique N-terminal extension used for peptidoglycan binding. Sci. Rep..

[323] Abdelkader K., Gutiérrez D., Grimon D., Ruas-Madiedo P., Lood C., Lavigne R., Safaan A., Khairalla A.S., Gaber Y., Dishisha T. (2020). Lysin LysMK34 of Acinetobacter baumannii Bacteriophage PMK34 Has a Turgor Pressure-Dependent Intrinsic Antibacterial Activity and Reverts Colistin Resistance. Appl. Environ. Microbiol..

[324] Zampara A., Sørensen M.C.H., Grimon D., Antenucci F., Vitt A.R., Bortolaia V., Briers Y., Brøndsted L. (2020). Exploiting phage receptor binding proteins to enable endolysins to kill Gram-negative bacteria. Sci. Rep..

[325] Briers Y., Walmagh M., Van Puyenbroeck V., Cornelissen A., Cenens W., Aertsen A., Oliveira H., Azeredo J., Verween G., Pirnay J.-P. (2014). Engineered Endolysin-Based “Artilysins” To Combat Multidrug-Resistant Gram-Negative Pathogens. MBio.

[326] Zampara A., Sørensen M.C.H., Grimon D., Antenucci F., Briers Y., Brøndsted L. (2018). Innolysins: A novel approach to engineer endolysins to kill Gram-negative bacteria. bioRxiv.

[327] Wan X., Hendrix H., Skurnik M., Lavigne R. (2021). Phage-based target discovery and its exploitation towards novel antibacterial molecules. Curr. Opin. Biotechnol..

[328] Fauconnier A. (2019). Phage Therapy Regulation: From Night to Dawn. Viruses.

[329] García R., Latz S., Romero J., Higuera G., García K., Bastías R. (2019). Bacteriophage Production Models: An Overview. Front. Microbiol..

[330] Górski A., Międzybrodzki R., Węgrzyn G., Jończyk-Matysiak E., Borysowski J., Weber-Dąbrowska B. (2020). Phage therapy: Current status and perspectives. Med. Res. Rev..

[331] Kleiner M., Bushnell B., Sanderson K.E., Hooper L.V., Duerkop B.A. (2020). Transductomics: Sequencing-based detection and analysis of transduced DNA in pure cultures and microbial communities. Microbiome.

[332] Kahn L.H., Bergeron G., Bourassa M.W., De Vegt B., Gill J., Gomes F., Malouin F., Opengart K., Ritter G.D., Singer R.S. (2019). From farm management to bacteriophage therapy: Strategies to reduce antibiotic use in animal agriculture. Ann. N. Y. Acad. Sci..

